# Event-Triggered Prescribed Performance Control for Maglev Systems Subject to Multiple Constraints

**DOI:** 10.3390/e28080934

**Published:** 2026-08-20

**Authors:** Chenglong Zhu, Xiaolong Chen, Xinming Guo, Wei Sun

**Affiliations:** 1 School of Mathematics and Systems Science, Liaocheng University, Liaocheng 252000, China; xiaolongchen1008@163.com (X.C.); sunwei@lcu.edu.cn (W.S.); 2 School of Cybersecurity, Northwestern Polytechnical University, Xi’an 710072, China; 3 Xinfa Group Co., Ltd., Liaocheng 252100, China; xinfagxm@163.com

**Keywords:** multiple constraints, maglev system, prescribed performance, dynamic gain, event-triggered control

## Abstract

Maglev trains are susceptible to various types of operational challenges, including track irregularities, load variations, and actuator faults. It is evident that these factors can compromise suspension performance and even pose a serious risk to operational safety. This paper proposes a prescribed performance event-triggered fault-tolerant control method for the electromagnetic suspension system of a maglev train subject to multiple constraints. A projection-based adaptive extended state observer is designed to estimate the unknown gain caused by actuator faults and load variations, as well as the external disturbance. In light of the disparity in upper and lower safety margins inherent to the suspension gap error, arising from track irregularities, an asymmetric prescribed performance function and an error transformation are devised to ensure that the gap tracking error perpetually complies with the asymmetric prescribed performance constraint. In addressing the issue of rapid variations in the suspension gap, the vertical velocity is also constrained through the implementation of prescribed performance, resulting in a joint constraint framework that encompasses both the gap tracking error and the vertical motion. A dynamic event-triggered mechanism has been incorporated into the backstepping design with a view to reducing unnecessary control updates under limited communication resources, while Zeno behavior has been excluded from the closed-loop system. Within this framework, a dynamic gain adjustment mechanism with an explicitly bounded rate of variation is further developed to achieve smoother gain adaptation. The uniform ultimate boundedness of all closed-loop signals is demonstrated through Lyapunov stability analysis under the prescribed multiple constraints. The efficacy of the proposed method is demonstrated through comparative simulation results.

## 1. Introduction

Maglev trains rely on electromagnetic forces to maintain contactless support between the vehicle body and the guideway. The principal advantages of maglev trains are reduced noise, minimized mechanical wear, and considerable potential for high-speed operation. The suspension system is a fundamental subsystem of a maglev train and its dynamic response exerts a direct influence on both running safety and ride comfort. The electromagnetic force depends nonlinearly on both the suspension gap and the coil current [1,2,3]. Extensive efforts have been devoted to nonlinear system control. Beyond continuous nonlinear systems, robustness analysis for controlled networks subject to disturbances has also attracted attention, including the computation of robust cycles in Boolean control networks [4]. Sliding-mode robust control, delay-dependent stabilization, and H∞ control for nonlinear impulsive systems have been extensively studied for uncertain nonlinear systems with input nonlinearities [5,6]. Observer-based admissibilization methods have also been developed for delayed degenerate jump systems, providing an alternative means of addressing unavailable state information and impulsive dynamics [7]. Finite-time and fault-tolerant control techniques under actuator faults and input constraints have also received considerable attention [8]. Beyond deterministic formulations, averaging principles for distribution-dependent slow-fast stochastic differential equations and state-feedback stabilization of stochastic nonlinear systems have also been investigated [9,10]. These works provide a useful theoretical basis for the development of maglev suspension control methods. From an information-theoretic perspective, the feedback control of maglev suspension systems can be interpreted as the transmission of state-dependent information from sensors to controllers and actuators. The efficiency and reliability of this information transfer under disturbances and faults directly affect the controller’s ability to preserve suspension safety and transient performance. For maglev systems, existing studies cover finite-time stabilization controller design and the application of finite-time stability theory to suspension dynamics [11,12]. Related studies on nonlinear vibration systems have investigated periodic and homoclinic responses of damped systems, providing useful theoretical tools for characterizing complex vibration behaviors [13,14,15]. However, most deterministic maglev models do not fully address external disturbances in practical operation. Macleod et al. [16] incorporated frequency-domain weighting into an LQR design to improve the suspension response under disturbances, although the use of a linearized model limits its ability to describe the intrinsic nonlinearity of the suspension system. With further consideration of disturbance factors such as track irregularities, a disturbance observer was employed in [17] for compensation under the assumption of a constant load. To address unknown nonlinearities and load disturbances in electromechanical systems, a neural adaptive funnel dynamic surface controller was developed in [18]. The method in [19] further enhanced closed-loop stability under uncertainty, but coordinated constraints on key variables such as the gap error and vertical velocity were not systematically characterized. Previous works have gradually expanded from deterministic control to address issues including disturbance rejection, track irregularity compensation, and load variation adaptation. However, in complex operating conditions, it remains necessary to address suspension gap safety, vertical vibration suppression, and transient performance requirements simultaneously.

In complex operating conditions, the performance of maglev systems must be maintained at both transient and steady-state levels in order to ensure stability. Prescribed performance control integrates the convergence rate, steady-state accuracy, and maximum overshoot into a time-varying performance function, and achieves system output constraints by employing a combination of barrier Lyapunov functions. This approach has gained significant traction in the field of high-precision control of uncertain nonlinear systems [20,21,22]. For high-speed maglev trains subject to track irregularities, a disturbance observer based prescribed performance controller was developed in [23]. A composite prescribed-performance backstepping controller incorporating a disturbance observer was developed for high-order nonlinear systems subject to bounded disturbances in [24]. These references use symmetric error constraints, where the positive and negative gap deviations share the same admissible boundary. Nonetheless, a conspicuous disparity exists between the upper and lower safety margins of the suspension air gap in practical engineering applications. An excessively small gap may cause contact between the electromagnet and the rail, whereas an excessively large gap may lead to insufficient attraction and suspension instability. Further considerations pertaining to track irregularities result in the imposition of more stringent requirements on asymmetric constraints. To accommodate different gap thresholds, asymmetric prescribed performance functions have been developed. Specifically, a piecewise asymmetric performance function was constructed in [25] to regulate the upper and lower output bounds independently; an asymmetric fixed time performance function was combined with sliding mode control in [26] to improve disturbance rejection; and the integration of asymmetric prescribed performance with anti-saturation control was reported in [27] to realize asymmetric gap constraints. To the best of the authors’ knowledge, extant prescribed performance studies on maglev systems principally focus on the suspension gap error, whereas systematic treatment of prescribed performance constraints on train vertical velocity remains rare. In practice, track irregularities, aerodynamic disturbances, and load fluctuations may excite pronounced vertical vibration of the vehicle body, which has a detrimental effect on ride comfort and may further amplify gap fluctuations. This paper sets out a hierarchical prescribed performance framework involving both the suspension gap and the vertical velocity. In this sense, the prescribed performance functions not only provide engineering safety envelopes but also act as an information-structuring mechanism. The admissible evolution of the tracking error and vertical velocity is encoded into time-varying constraint sets. This enables the controller to distinguish between acceptable transient deviations and informative error growth caused by disturbances, load variations, or actuator faults.

The long-term operation of maglev systems is characterized by a number of practical concerns, with actuator faults representing a significant challenge. Event-triggered fault-tolerant control for uncertain nonlinear systems has been investigated in [28,29,30]. Since load variations, disturbances, and fault evolution may occur on different time scales, averaging principles for mixed slow-fast stochastic differential equations provide useful analytical tools for multiscale stochastic dynamics [31]. Typical actuator faults include partial loss of effectiveness, bias faults, and stuck faults. A constrained active fault-tolerant control scheme based on active fault diagnosis and interpolation optimization was proposed in [32] to address component and actuator faults under state and input constraints. A dynamic event-triggered fault-tolerant strategy was developed in [33] to reduce the communication load between the controller and the actuator while maintaining fault-tolerant performance. Output feedback compensation for actuator faults was studied in [34,35] when full state measurements are unavailable. In maglev train suspension systems, actuator faults directly affect the electromagnetic force and may threaten suspension safety. To address this issue, different suspension submodels were built in [36] for different fault severities, and bumpless switching between fault modes was achieved. In [37], Nussbaum gains and fuzzy neural networks were used to handle unknown control directions caused by actuator stuck faults. It should be noted that the majority of these results are developed under the assumption of constant suspension mass, which may not always be an accurate representation of real-world conditions. In practical terms, load variations have the capacity to effect significant changes in the equivalent mass and thereby to alter the gain of the electromagnetic force channel. In the event of such variations occurring in conjunction with actuator faults, the resulting gain mismatch may lead to large suspension gap fluctuations, violation of prescribed performance constraints, or even loss of stable suspension. These engineering considerations necessitate a fault-tolerant control design that also incorporates load adaptation.

Conventional control implementations characteristically depend on either periodic sampling or continuous time sampling [38]. The implementation of Lebesgue nonuniform sampling has been proposed as a means to enhance the efficacy of control strategies [39]. It is imperative to emphasize that the modification of control inputs exclusively in instances of necessity has the capacity to minimize superfluous data collection whilst conserving valuable onboard communication assets. The potential of event-triggered control to eliminate redundant sampling and reduce onboard communication burden has led to its emergence as a research focus in the domain of maglev control systems. This viewpoint is closely related to information-theoretic control, in which the key issue is not transmitting all sampled data, but rather transmitting only the information that is sufficiently relevant to closed-loop performance. In event-triggered control, the triggering condition acts as an information filter, suppressing redundant measurements or control updates while transmitting significant changes in the control signal to the actuator. Consequently, the proposed event-triggered mechanism can be considered a communication-efficient strategy for transferring information in constrained maglev suspension control. Event-triggered adaptive control can reduce the update frequency of control signals while preserving closed-loop stability, thereby lowering the computational burden on embedded controllers [40]. A dynamic event-triggered strategy with time varying thresholds was adopted in [41] to reduce communication consumption while maintaining output bounds for the suspension gap. A dynamic event-triggered sliding mode controller based on stability conditions was proposed in [42] to reduce computation while ensuring robust stability. Many existing event-triggered schemes use fixed gains, which do not adapt the control weight to the evolution of the tracking error. Dynamic gain mechanisms enable adaptive updates of control parameters at triggering instants [43,44,45], alleviating the limitations of constant gains. Nevertheless, existing event-triggered strategies with dynamic gains fail to bound gain variations, hindering their application in maglev systems. Meanwhile, unrestrained dynamic adjustment tends to induce persistent oscillations in system states and further increase triggering times, making it impossible to simultaneously satisfy the dual requirements of saving communication resources and ensuring safe actuator operation. Motivated by the dynamic gain event-triggered idea in [43], this paper constructs a rate-constrained dynamic gain update scheme. Within the confines of the prescribed performance constraints imposed on the air gap and vertical velocity, a rate-constrained dynamic gain update law is formulated to address actuator faults while concurrently reducing communication demands. The employment of a saturation operator serves to impose a limit on the maximum rate of gain variation, thereby ensuring continuous and smooth adaptive adjustment of the gain.

The main contributions of this paper are as follows:

1. Different from the nominal model [11] and the linearized approximation [16], this paper explores the combined effects of track irregularity excitation, load variation, and actuator faults in high-speed maglev operation. A nonlinear dynamic model of the maglev train suspension system is established, and a projection-based adaptive extended state observer is developed to estimate the disturbance arising from multiple coupled sources and the unknown control gain. The resulting model offers a more accurate reflection of practical operating conditions and supports suspension stability and control accuracy when several adverse factors coexist.
2. In contrast to the symmetric prescribed performance [23] and the single-variable asymmetric constraints [25], in this study, a novel dual-layer prescribed-performance constraint framework is developed for the levitation gap error and the vertical velocity. This framework is based on the engineering characteristics of unequal upper and lower safety margins of the levitation gap, as well as the need to suppress vertical motion. The proposed method features independent adjustment of overshoot and steady-state accuracy bounds for bidirectional suspension gap deviations and leverages velocity constraints to attenuate severe carbody vibrations, thereby achieving a coordinated guarantee of operational safety and passenger comfort.
3. In distinction to the constant gain [11] and the dynamic gain mechanism [43], this paper proposes a rate-constrained dynamic gain by skillfully constructing a saturation-based update law to facilitate smooth adaptive gain adjustment. In scenarios characterized by numerous constraints, encompassing multi-source disturbances, constrained communication resources, and stipulated performance specifications, it has been theoretically substantiated that the closed-loop system is uniformly ultimately bounded and devoid of Zeno behavior.

## 2. Problem Formulation and Control Objectives

### 2.1. Maglev System Model with Actuator Faults

**Figure 1:**
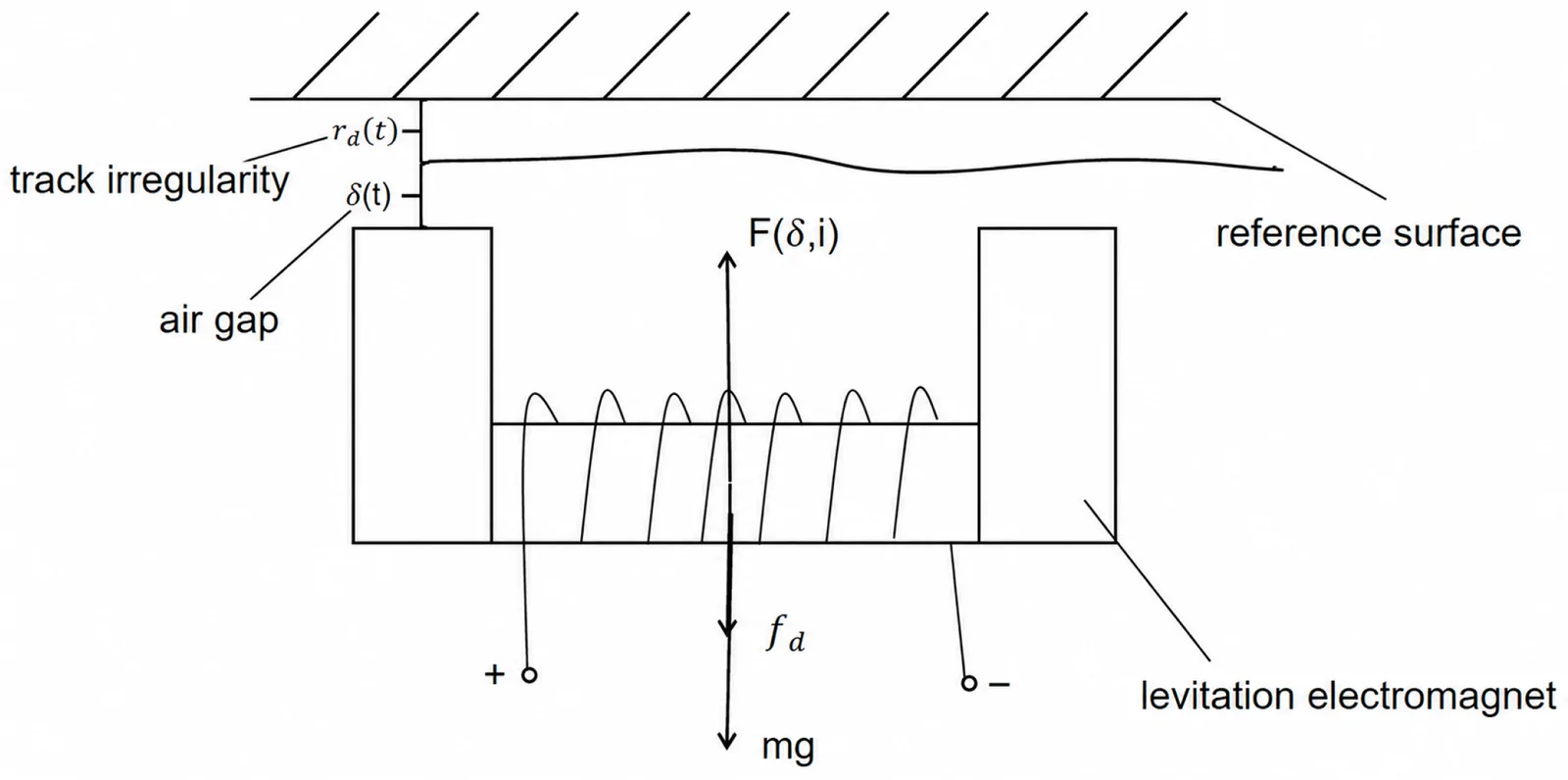
Force diagram of the suspension electromagnet.

Experiments indicate that the design of the levitation controller can be simplified to a single electromagnet levitation problem within a certain range because of the structural decoupling function of the levitation frame [46]. The schematic of the maglev train suspension system with track irregularity is shown in Figure 1.

Neglecting magnetic flux leakage and the reluctance of the electromagnet core and rail [47], the effective air gap reluctance between the electromagnet and the rail can be expressed as

$$R_{g}\left(\delta \right)=\frac{2\delta }{\mu_{0}S}$$

The coil inductance of the electromagnet is inversely proportional to the air gap reluctance and can be written as

$$L\left(\delta \right)=\frac{N^{2}}{R_{g}\left(\delta \right)}=\frac{\mu_{0}N^{2}S}{2\delta }$$

where *N* denotes the number of turns of the electromagnet coil.

The magnetic field energy stored in the system is given by

$$W_{m}\left(i,\delta \right)=\frac{1}{2}L\left(\delta \right)i^{2}=\frac{\mu_{0}N^{2}Si^{2}}{4\delta }$$

where i(t) is the excitation current in the coil. The magnitude of the electromagnetic attraction is given by

(1) $$F\left(i,\delta \right)=-\frac{\partial W_{m}\left(i,\delta \right)}{\partial \delta }=\frac{\mu_{0}N^{2}S}{4}\cdot \frac{i^{2}}{\delta^{2}}.$$

Considering the influence of track irregularities, the guideway may exhibit vertical irregularities caused by structural deformation and installation errors during train operation. Let $r_{d}\left(t\right)$ denote the vertical displacement due to track irregularity. The midpoint deformation of a simply supported guideway beam is usually approximated by a sinusoidal function [48]:

$$r_{d}\left(t\right)=Z_{\max}\sin\left(\frac{\pi vt}{L}+\pi \right)$$

where $Z_{\max}$ is the maximum midpoint deformation of the guideway, *v* is the train speed and *L* is the span of the simply supported beam.

Define the generalized displacement $x_{1}\left(t\right)$ as the vertical displacement of the electromagnet pole surface relative to a reference plane. Then, the actual suspension gap satisfies

(2) $$\delta \left(t\right)=x_{1}\left(t\right)-r_{d}\left(t\right)$$

For a single suspension electromagnet, the forces acting on it include the downward gravity mg, the external vertical disturbance force $f_{d}\left(t\right)$ and the upward electromagnetic attraction F(i,δ). According to Newton’s second law, the vertical dynamics of the electromagnet is

(3) $$m{\ddot{x}}_{1}\left(t\right)=mg+f_{d}\left(t\right)-F\left(i,\delta \right)$$

where *m* is the equivalent total mass of the load, suspension frame and electromagnet, and $g=9.8\;m/s^{2}$ is the gravitational acceleration.

Considering load variations during practical train operation, the mass *m* is bounded as

(4) $$0<m_{\min}\leq m\leq m_{\max}$$

where $m_{\min}$ and $m_{\max}$ are the known total masses under no-load and full-load conditions, respectively.

Considering actuator faults, let $i^{2}=u\left(t\right)$ be the control input. The following fault model is adopted:

(5) $$u_{a}\left(t\right)=\mu \left(t\right)u\left(t\right)+u_{b}\left(t\right)$$

where u(t) is the nominal control input, μ(t) is the actuator effectiveness coefficient, $u_{b}\left(t\right)$ is the additive bias fault and $u_{a}\left(t\right)$ is the actual control input. Substituting $u_{a}\left(t\right)$ into (1) yields the electromagnetic attraction under actuator faults. Substituting it further into (3) gives the fault affected dynamic model as

(6) $$m{\ddot{x}}_{1}\left(t\right)=mg+f_{d}\left(t\right)-\frac{\mu_{0}N^{2}S}{4\delta^{2}}\left[\mu \left(t\right)u\left(t\right)+u_{b}\left(t\right)\right]$$

After rearrangement, the nonlinear model of the maglev system with actuator faults is obtained as

(7) $$\left\{\begin{array}{c} {\dot{x}}_{1}\left(t\right)=x_{2}\left(t\right),\\ {\dot{x}}_{2}\left(t\right)=-\frac{\mu (t)}{m}\frac{b}{{\left(x_{1}-r_{d}\right)}^{2}}u\left(t\right)+g+D_{f}\left(t\right),\\ y=x_{1}\left(t\right). \end{array}\right.$$

where $x_{2}\left(t\right)$ is the vertical velocity, and *y* is the system output. Here, $b=\frac{\mu_{0}N^{2}S}{4}$ is the equivalent coefficient of the electromagnetic attraction, and $D_{f}\left(t\right)=\frac{f_{d}\left(t\right)}{m}-\frac{bu_{b}\left(t\right)}{m{\left(x_{1}-r_{d}\right)}^{2}}$ is the lumped disturbance.

### 2.2. Assumptions

**Assumption** **1** ([43])**.** *There exist unknown constants $\mu_{L}>0$ and $u_{b}>0$ such that*

(8) $$0<\mu_{L}\leq \mu \left(t\right)\leq 1,∥u_{b}\left(t\right)∥\leq u_{b}$$

*and the actuator fault is slowly time-varying.*

**Assumption** **2** ([17])**.** *The lumped disturbance and its first-order derivative are bounded; There exists a constant $D_{f0}>0$ such that*

(9) $$|{\dot{D}}_{f}\left(t\right)|\;\leq D_{f0}$$

### 2.3. Control Objectives

The objective of this paper is to design a two-layer prescribed performance dynamic event-triggered fault-tolerant control strategy such that all closed-loop signals are uniformly ultimately bounded, the dynamic event-triggered mechanism is free of Zeno behavior, and the following constraints are satisfied.

(1) Asymmetric gap safety constraint: The tracking error $e_{1}\left(t\right)$ satisfies

(10) $$-\delta_{2}\left(t\right)<e_{1}\left(t\right)<\delta_{1}\left(t\right),\;\forall t\geq 0,$$

where $\delta_{1}\left(t\right)$ and $\delta_{2}\left(t\right)$ are the allowable upper and lower bounds of the suspension gap tracking error, respectively.

(2) Vertical velocity comfort constraint: The vertical velocity $x_{2}\left(t\right)$ satisfies

(11) $$\left|x_{2}\left(t\right)\right|<\rho_{2}\left(t\right),\;\forall t\geq 0,$$

where $\rho_{2}\left(t\right)$ is the prescribed performance function specifying its admissible variation range.

(3) Dynamic gain and rate constraint: The dynamic gain $k_{2}\left(t\right)$ satisfies

(12) $$0\leq {\dot{k}}_{2}\left(t\right)\leq {\bar{r}}_{k},\;k_{2}\left(t\right)\geq k_{20},\;\forall t\geq 0,$$

where ${\bar{r}}_{k}$ is the prescribed upper bound on the variation rate of the dynamic gain, and $k_{20}$ is its initial value.

**Remark** **1.** *The proposed asymmetric gap constraint is a generalized formulation. When $\delta_{1}\left(t\right)=\delta_{2}\left(t\right)$, it degenerates into the symmetric gap constraint adopted in [23]. Therefore, the constraint in [23] can be regarded as a special case of the proposed one, while the asymmetric formulation provides greater flexibility for practical maglev systems with unequal upper and lower safety margins.*

**Remark** **2.** *The dynamic gain-rate constraint is a vital component in ensuring system stability. It functions by preventing excessively rapid gain adaptation and abrupt control-input variations, which is beneficial for actuator operation. The three constraints are integrated to ensure that the proposed design incorporates suspension safety, ride comfort, and actuator-friendly fault-tolerant control.*

## 3. Main Results

### 3.1. Adaptive Extended State Observer

The estimation of the lumped disturbance is achieved through its consideration as a virtual system state, represented by $x_{3}=D_{f}\left(t\right)$. This state is subsequently incorporated into an augmented system to facilitate analysis. From (7), the extended system can be obtained as

(13) $$\left\{\begin{array}{cc} {\dot{x}}_{1} & =x_{2},\\ {\dot{x}}_{2} & ={\hat{\theta }}_{\mu }\phi \left(x_{1},u,r_{d}\right)+g+x_{3}-{\tilde{\theta }}_{\mu }\phi \left(x_{1},u,r_{d}\right),\\ {\dot{x}}_{3} & ={\dot{D}}_{f}\left(t\right),\\ y & =x_{1}. \end{array}\right.$$

where $\theta_{\mu }\left(t\right)=\frac{\mu (t)}{m}$, $f\left(x_{1},r_{d}\right)=-\frac{1}{{\left(x_{1}-r_{d}\right)}^{2}}$, $\phi \left(x_{1},u,r_{d}\right)=bf\left(x_{1},r_{d}\right)u$, ${\hat{\theta }}_{\mu }$ is the estimate of $\theta_{\mu }$ and the parameter estimation error is ${\tilde{\theta }}_{\mu }={\hat{\theta }}_{\mu }-\theta_{\mu }$. Design the extended state observer as

(14) $$\left\{\begin{array}{cc} {\dot{\hat{x}}}_{1} & ={\hat{x}}_{2}+3\omega \left(y-{\hat{x}}_{1}\right),\\ {\dot{\hat{x}}}_{2} & ={\hat{\theta }}_{\mu }\phi \left(x_{1},u,r_{d}\right)+g+{\hat{x}}_{3}+3\omega^{2}\left(y-{\hat{x}}_{1}\right),\\ {\dot{\hat{x}}}_{3} & =\omega^{3}\left(y-{\hat{x}}_{1}\right), \end{array}\right.$$

where ω>0 is the observer bandwidth. Introduce the estimation errors

(15) $${\tilde{x}}_{1}=x_{1}-{\hat{x}}_{1},\;{\tilde{x}}_{2}=x_{2}-{\hat{x}}_{2},\;{\tilde{x}}_{3}=x_{3}-{\hat{x}}_{3},$$

Design the following scaled error transformation:

(16) $$\varepsilon ={\left[\begin{array}{ccc} \varepsilon_{1} & \varepsilon_{2} & \varepsilon_{3} \end{array}\right]}^{T}={\left[\begin{array}{ccc} {\tilde{x}}_{1} & {\tilde{x}}_{2}/\omega & {\tilde{x}}_{3}/\omega^{2} \end{array}\right]}^{T}.$$

From (13) and (14), one has

(17) $$\begin{array}{cc} {\dot{\tilde{x}}}_{1} & ={\tilde{x}}_{2}-3\omega {\tilde{x}}_{1}, \end{array}$$

(18) $$\begin{array}{cc} {\dot{\tilde{x}}}_{2} & ={\tilde{x}}_{3}-3\omega^{2}{\tilde{x}}_{1}-{\tilde{\theta }}_{\mu }\phi \left(x_{1},u,r_{d}\right), \end{array}$$

(19) $$\begin{array}{cc} {\dot{\tilde{x}}}_{3} & ={\dot{D}}_{f}\left(t\right)-\omega^{3}{\tilde{x}}_{1}. \end{array}$$

Furthermore, according to the definition of the scaled errors, it follows that

(20) $$\begin{array}{cc} {\dot{\varepsilon }}_{1} & =\omega (\varepsilon_{2}-3\varepsilon_{1}), \end{array}$$

(21) $$\begin{array}{cc} {\dot{\varepsilon }}_{2} & =\omega \left(\varepsilon_{3}-3\varepsilon_{1}\right)-\frac{{\tilde{\theta }}_{\mu }\phi }{\omega }, \end{array}$$

(22) $$\begin{array}{cc} {\dot{\varepsilon }}_{3} & =-\omega \varepsilon_{1}+\frac{{\dot{D}}_{f}\left(t\right)}{\omega^{2}}. \end{array}$$

Thus, the compact matrix-vector form is obtained as

(23) $$\dot{\varepsilon }=\omega A\varepsilon +C_{1}\left(-\frac{{\tilde{\theta }}_{\mu }\phi }{\omega }\right)+C_{3}\left(\frac{{\dot{D}}_{f}\left(t\right)}{\omega^{2}}\right).$$

where

(24) $$A=\left[\begin{array}{ccc} -3 & 1 & 0\\ -3 & 0 & 1\\ -1 & 0 & 0 \end{array}\right],\;C_{1}=\left[\begin{array}{c} 0\\ 1\\ 0 \end{array}\right],\;C_{3}=\left[\begin{array}{c} 0\\ 0\\ 1 \end{array}\right].$$

It can be verified that matrix A is Hurwitz [49].

Design the projection-based adaptive law as

(25) $${\dot{\hat{\theta }}}_{\mu }={\mathrm{Proj}}_{{\hat{\theta }}_{\mu }}\left(\Gamma \left[-\frac{\phi {\tilde{x}}_{1}}{\omega \left(1+\phi^{2}\left(t\right)\right)}-\sigma {\hat{\theta }}_{\mu }\right]\right).$$

where Γ>0 and σ>0. From Assumption 1, one has

(26) $$\theta_{\mu \min}\leq \theta_{\mu }\leq \theta_{\mu \max},\;\theta_{\mu \min}=\frac{\mu_{L}}{m_{\max}},\;\theta_{\mu \max}=\frac{1}{m_{\min}}.$$

Following the projection operator update for ${\hat{\theta }}_{\mu }$ in [50] and with ${\hat{\theta }}_{\mu }\left(0\right)\in \left[\theta_{\mu \min},\theta_{\mu \max}\right]$, it follows that

(27) $${\hat{\theta }}_{\mu }\left(t\right)\in \left[\theta_{\mu \min},\theta_{\mu \max}\right],\;\forall t\geq 0,$$

and for any adaptive function τ, the following property holds:

(28) $${\tilde{\theta }}_{\mu }\left(\Gamma^{-1}\mathrm{Proj}\left(\Gamma \tau \right)-\tau \right)\leq 0.$$

**Theorem** **1.** *Under Assumptions 1 and 2, with the extended state observer given in (14) and the adaptive update law constructed in (25), both the observation error ε and the parameter estimation error ${\tilde{\theta }}_{\mu }$ are guaranteed to be uniformly ultimately bounded.*

**Proof.**  Construct the Lyapunov function

(29) $$V_{o}=\frac{1}{2}\varepsilon^{T}P\varepsilon +\frac{1}{2}\Gamma^{-1}{\tilde{\theta }}_{\mu }^{2}.$$

where *P* denotes a symmetric positive definite matrix. Differentiating $V_{o}$ yields

(30) $${\dot{V}}_{o}=\varepsilon^{T}P\dot{\varepsilon }+\Gamma^{-1}{\tilde{\theta }}_{\mu }{\dot{\hat{\theta }}}_{\mu }.$$

Since *A* is Hurwitz, for any positive definite matrix *Q*, the Lyapunov algebraic equation $A^{T}P+PA=-Q$ admits a unique positive definite solution [50]

(31) $$P=\left[\begin{array}{ccc} 2 & -1 & -2\\ -1 & 2 & -1\\ -2 & -1 & 8 \end{array}\right].$$

Substituting the error system (23) gives

(32) $${\dot{V}}_{o}=-\omega {∥\varepsilon ∥}^{2}-\frac{{\tilde{\theta }}_{\mu }\phi }{\omega }\varepsilon^{T}PC_{1}+\frac{{\dot{D}}_{f}\left(t\right)}{\omega^{2}}\varepsilon^{T}PC_{3}+\Gamma^{-1}{\tilde{\theta }}_{\mu }{\dot{\hat{\theta }}}_{\mu }.$$

From the analytical solution of the matrix *P*, $PC_{1}$ is the second column of *P*, namely, $PC_{1}={\left[-1,\;2,\;-1\right]}^{T}$. Therefore,

(33) $$\varepsilon^{T}PC_{1}=-\varepsilon_{1}+2\varepsilon_{2}-\varepsilon_{3}=-{\tilde{x}}_{1}+\left(2\varepsilon_{2}-\varepsilon_{3}\right).$$

We obtain

(34) $${\dot{V}}_{o}=-\omega {∥\varepsilon ∥}^{2}+\frac{{\tilde{\theta }}_{\mu }\phi {\tilde{x}}_{1}}{\omega }-\frac{{\tilde{\theta }}_{\mu }\phi \left(2\varepsilon_{2}-\varepsilon_{3}\right)}{\omega }+\frac{{\dot{D}}_{f}\left(t\right)}{\omega^{2}}\varepsilon^{T}PC_{3}+\Gamma^{-1}{\tilde{\theta }}_{\mu }{\dot{\hat{\theta }}}_{\mu }.$$

From (25) and (28), one has

(35) $$\Gamma^{-1}{\tilde{\theta }}_{\mu }{\dot{\hat{\theta }}}_{\mu }\leq {\tilde{\theta }}_{\mu }\left(-\frac{\phi {\tilde{x}}_{1}}{\omega (1+\phi^{2}\left(t\right))}-\sigma {\hat{\theta }}_{\mu }\right).$$

Substituting the above inequality and combining the main cross terms yields

(36) $${\dot{V}}_{o}\leq -\omega {∥\varepsilon ∥}^{2}+\frac{{\tilde{\theta }}_{\mu }\phi^{3}{\tilde{x}}_{1}}{\omega (1+\phi^{2}\left(t\right))}-\frac{{\tilde{\theta }}_{\mu }\phi \left(2\varepsilon_{2}-\varepsilon_{3}\right)}{\omega }+\frac{{\dot{D}}_{f}\left(t\right)}{\omega^{2}}\varepsilon^{T}PC_{3}-\sigma {\tilde{\theta }}_{\mu }{\hat{\theta }}_{\mu }.$$

Via the Cauchy inequality, this yields $|2\varepsilon_{2}-\varepsilon_{3}|\;\leq \sqrt{5}∥\varepsilon ∥$. Using the boundedness of the projection operator, there exists a constant ${\tilde{\theta }}_{\mu M}>0$ such that $|{\tilde{\theta }}_{\mu }|\;\leq {\tilde{\theta }}_{\mu M}$. Since $|{\tilde{x}}_{1}|\;\leq ∥\varepsilon ∥$, one obtains

(37) $$|\frac{{\tilde{\theta }}_{\mu }\phi^{3}{\tilde{x}}_{1}}{\omega (1+\phi^{2}\left(t\right))}|\;\leq \frac{{\tilde{\theta }}_{\mu M}\left|\phi \right|∥\varepsilon ∥}{\omega },$$

(38) $$|\frac{{\tilde{\theta }}_{\mu }\phi \left(\varepsilon_{3}-2\varepsilon_{2}\right)}{\omega }|\;\leq \frac{\sqrt{5}{\tilde{\theta }}_{\mu M}\left|\phi \right|∥\varepsilon ∥}{\omega }.$$

According to the definition of ϕ, under bounded test inputs and with a strictly positive lower bound of the actual gap, there exists $\phi_{M}>0$ such that $\left|\phi (t)\right|\leq \phi_{M}$. Combining the above terms and applying Young’s inequality gives

(39) $$\frac{\left(1+\sqrt{5}\right){\tilde{\theta }}_{\mu M}\left|\phi \right|∥\varepsilon ∥}{\omega }\leq \frac{1}{4}{∥\varepsilon ∥}^{2}+\frac{{\left(1+\sqrt{5}\right)}^{2}{\tilde{\theta }}_{\mu M}^{2}\phi_{M}^{2}}{\omega^{2}},$$

By Assumption 2, inequality bounding similar to the (39),

(40) $$|\frac{{\dot{D}}_{f}\left(t\right)}{\omega^{2}}\varepsilon^{T}PC_{3}|\;\leq \frac{1}{4}{∥\varepsilon ∥}^{2}+\frac{D_{f0}^{2}{∥PC_{3}∥}^{2}}{\omega^{4}}.$$

In addition, one has

(41) $$-\sigma {\tilde{\theta }}_{\mu }{\hat{\theta }}_{\mu }=-\sigma {\tilde{\theta }}_{\mu }^{2}-\sigma \theta_{\mu }{\tilde{\theta }}_{\mu }\leq -\frac{\sigma }{2}{\tilde{\theta }}_{\mu }^{2}+\frac{\sigma }{2}\theta_{\mu \max}^{2}.$$

Combining the above results gives

(42) $${\dot{V}}_{o}\leq -\left(\omega -\frac{1}{2}\right){∥\varepsilon ∥}^{2}-\frac{\sigma }{2}{\tilde{\theta }}_{\mu }^{2}+\sigma_{o},$$

where

(43) $$\sigma_{o}=\frac{{\left(1+\sqrt{5}\right)}^{2}{\tilde{\theta }}_{\mu M}^{2}\phi_{M}^{2}}{\omega^{2}}+\frac{D_{f0}^{2}{∥PC_{3}∥}^{2}}{\omega^{4}}+\frac{\sigma }{2}\theta_{\mu \max}^{2}.$$

Using the eigenvalue inequalities of the Lyapunov function, choose

(44) $$\lambda_{o}=\min\left\{\frac{2\omega -1}{\lambda_{\max}\left(P\right)},\;\sigma \Gamma \right\}>0,$$

Thus,

(45) $${\dot{V}}_{o}\leq -\lambda_{o}V_{o}+\sigma_{o}.$$

Since $\sigma_{o}$ is a globally bounded constant, $V_{o}$ exponentially converges to a bounded set. Hence, the scaled error ε and the parameter estimation error ${\tilde{\theta }}_{\mu }$ are uniformly ultimately bounded. □

**Remark** **3.** *From (43), $\sigma_{o}$ contains the error term induced by the variation rate of the extended disturbance. This term is inversely proportional to the fourth power of the observer bandwidth ω. Under circumstances where the other parameters are fixed, a suitable increase in the value of ω can effectively mitigate the deleterious effect of this term on the ultimate bound of the observation error.*

### 3.2. Dynamic Event-Triggered Fault-Tolerant Controller Design

To ensure that the suspension-gap error satisfies the prescribed transient and steady-state constraints in the presence of faults and disturbances, the following performance function is selected:

(46) $$\rho_{1}\left(t\right)=\left(\rho_{10}-\rho_{1\infty }\right)e^{-l_{1}t}+\rho_{1\infty },$$

where $\rho_{10}>\rho_{1\infty }>0$, and $l_{1}>0$. Here, $\rho_{10}$ determines the initial width of the prescribed performance envelope and should be selected to satisfy the initial feasibility condition. The parameter $\rho_{1\infty }$ specifies the limiting width of the performance envelope and therefore determines the steady state tracking accuracy. The parameter $l_{1}$ governs the contraction rate of the envelope. Given the desired suspension gap $\delta_{0}$, define the desired generalized displacement trajectory as $x_{1d}\left(t\right)=\delta_{0}+r_{d}\left(t\right)$ and define the tracking error and the normalized error as

(47) $$e_{1}\left(t\right)=x_{1}\left(t\right)-x_{1d}\left(t\right),\;\eta_{1}=\frac{e_{1}}{\rho_{1}\left(t\right)}.$$

To satisfy the requirement of the logarithmic barrier function, let $\delta_{1}\left(t\right)={\bar{\delta }}_{1}\rho_{1}\left(t\right)$ and $\delta_{2}\left(t\right)={\underline{\delta }}_{1}\rho_{1}\left(t\right)$, where ${\bar{\delta }}_{1}$ and ${\underline{\delta }}_{1}$ are the positive-direction and negative-direction constraint coefficients, respectively. An error transformation is introduced to map the asymmetric constraint interval into a standard bounded interval. Define

(48) $$\xi_{1}=\frac{2\eta_{1}-\left({\bar{\delta }}_{1}-{\underline{\delta }}_{1}\right)}{{\bar{\delta }}_{1}+{\underline{\delta }}_{1}}.$$

For $|\xi_{1}|<1$, the following derivation is obtained:

$$\begin{array}{cc} \; & -1<\frac{2\eta_{1}-\left({\bar{\delta }}_{1}-{\underline{\delta }}_{1}\right)}{{\bar{\delta }}_{1}+{\underline{\delta }}_{1}}<1 \end{array}$$

Substituting $\eta_{1}=\frac{e_{1}}{\rho_{1}\left(t\right)}$ and applying equivalent transformations, multiplying both sides by ${\bar{\delta }}_{1}+{\underline{\delta }}_{1}$ gives:

$$\begin{array}{c} -\left({\bar{\delta }}_{1}+{\underline{\delta }}_{1}\right)<\frac{2e_{1}}{\rho_{1}\left(t\right)}-\left({\bar{\delta }}_{1}-{\underline{\delta }}_{1}\right)<{\bar{\delta }}_{1}+{\underline{\delta }}_{1} \end{array}$$

After further rearrangement, one obtains:

$$\begin{array}{c} -{\underline{\delta }}_{1}\rho_{1}\left(t\right)<e_{1}\left(t\right)<{\bar{\delta }}_{1}\rho_{1}\left(t\right) \end{array}$$

Therefore, $|\xi_{1}|<1$ is equivalent to (10).

The vertical velocity $x_{2}\left(t\right)$ is directly related to suspension stability and ride quality. Excessive velocity fluctuations may amplify gap deviations and aggravate vehicle body vibration. Therefore, a prescribed performance constraint is further imposed on $x_{2}\left(t\right)$ in addition to the gap constraint. Choose the performance function

(49) $$\rho_{2}\left(t\right)=\left(\rho_{20}-\rho_{2\infty }\right)e^{-l_{2}t}+\rho_{2\infty },$$

where $\rho_{20}>\left|x_{2}\left(0\right)\right|$, $\rho_{2\infty }>0$ and $l_{2}>0$. Define

(50) $$\eta_{2}=\frac{x_{2}}{\rho_{2}\left(t\right)}.$$

Then, $|\eta_{2}|\;<1$ is equivalent to (11).

Construct the logarithmic barrier function

(51) $$V_{1}=\frac{1}{2}\ln\frac{1}{1-\xi_{1}^{2}}.$$

From (48), one has

(52) $${\dot{\xi }}_{1}=\frac{2}{\left({\bar{\delta }}_{1}+{\underline{\delta }}_{1}\right)\rho_{1}}\left(x_{2}-{\dot{x}}_{1d}-\eta_{1}{\dot{\rho }}_{1}\right).$$

Design the virtual controller as

(53) $$\alpha_{1}={\dot{x}}_{1d}+\eta_{1}{\dot{\rho }}_{1}-\frac{{\bar{\delta }}_{1}+{\underline{\delta }}_{1}}{2}k_{1}\rho_{1}\xi_{1},$$

where $k_{1}>0$ is a design parameter. Based on the above virtual controller design, the continuous domain fault-tolerant control law is first constructed as

(54) $$\begin{array}{cc} u^{*}\left(t\right)=\frac{1}{{\hat{\theta }}_{\mu }bf\left(x_{1},r_{d}\right)}[ & -g-{\hat{x}}_{3}+\eta_{2}{\dot{\rho }}_{2}-k_{2}\left(t\right)\rho_{2}\eta_{2}\\ \; & -\frac{2\rho_{2}^{2}\left(1-\eta_{2}^{2}\right)}{\left({\bar{\delta }}_{1}+{\underline{\delta }}_{1}\right)\rho_{1}\left(1-\xi_{1}^{2}\right)}\xi_{1}-\kappa_{f}\mathrm{sgn}\left(\eta_{2}\right)], \end{array}$$

where $\kappa_{f}>0$ and $k_{2}\left(t\right)$ is the rate-constrained dynamic gain to be designed. Define the monotonically increasing time sequence ${\left\{t_{i}\right\}}_{i=0}^{\infty }$. For $t\in [t_{i},t_{i+1})$, the control input is generated by a zero-order hold as

(55) $$u\left(t\right)=u\left(t_{i}\right),\;\forall t\in \left[t_{i},t_{i+1}\right).$$

At each triggering instant, one has

(56) $$u\left(t_{i}\right)=v\left(t_{i}\right),\;e_{u}\left(t_{i}\right)=0.$$

The next triggering instant is determined when either of the following conditions is satisfied:

(57) $$\left\{\begin{array}{cc} \mathrm{Event}\;1:\; & \lim_{s\to t_{i}^{+}}\mathrm{sgn}\left(x_{2}\left(s\right)\right)\neq \lim_{s\to t_{i}^{-}}\mathrm{sgn}\left(x_{2}\left(s\right)\right)\;\mathrm{or}\\ \; & \lim_{s\to t_{i}^{+}}\mathrm{sgn}\left(x_{2}\left(s\right)\right)=\lim_{s\to t_{i}^{-}}\mathrm{sgn}\left(x_{2}\left(s\right)\right)\neq \mathrm{sgn}\left(x_{2}\left(t_{i}\right)\right),\\ \mathrm{Event}\;2:\; & |e_{u}\left(t\right)|\geq \Phi (|v(t)|)|u(t)|+\bar{e}. \end{array}\right.$$

where $e_{u}\left(t\right)≜v\left(t\right)-u\left(t\right)$ denotes the event triggering error, Φ(|v(t)|):[0,∞)→[0,1) is a monotonically increasing function satisfying Φ(0)=0, $\bar{e}>0$ is a positive design parameter and $t_{i}\left(i\in Z^{+}\right)$ denotes the controller update instant. One possible choice is

(58) $$\Phi \left(|v(t)|\right)=\tanh\left(\frac{\left|v(t)\right|}{M}\right),\;M>0.$$

Construct the enhanced continuous virtual control law as

(59) $$v\left(t\right)=\left(1+\rho_{r}\right)u^{*}\left(t\right)+\bar{e}\mathrm{sgn}\left(x_{2}\right),$$

where $\rho_{r}\in \left[1,2\right)$ and $\bar{e}>0$.

**Remark** **4.** *The parameter M regulates the trade-off between triggering frequency and control performance in the dynamic threshold (58). For a fixed |v|>0, a smaller M increases Φ(|v|), allowing a larger triggering error and generally reducing control updates, whereas a larger M yields a stricter threshold and more frequent updates. Moreover, Event 1 updates the controller whenever the sign of the vertical velocity changes, preventing the zero-order hold input and the sign-dependent compensation term from becoming inconsistent with the actual motion direction. Thus, Event 1 handles velocity reversals, while Event 2 responds to the accumulated input error.*

### 3.3. Rate-Constrained Dynamic Gain Design

To reduce the conservatism of fixed gains, dynamic gain control is widely used. However, discrete event-triggered updates may cause instantaneous gain jumps, and the resulting current shocks may damage the suspension electromagnet. Therefore, $k_{2}$ is designed as a rate-constrained dynamic gain $k_{2}\left(t\right)$. Define the monitoring time sequence for the dynamic gain as

(60) $$t_{j}^{k}=j\iota_{k},\;j=0,1,2,\ldots ,$$

where $\iota_{k}>0$. Let the initial gain be

(61) $$k_{2}\left(0\right)=k_{20}>0.$$

Define the monitoring vector

(62) $$\eta_{k}\left(t\right)={\left[\begin{array}{cc} \xi_{1}\left(t\right) & \eta_{2}\left(t\right) \end{array}\right]}^{T}.$$

Choose a class-$K_{\infty }$ monitoring function $\Lambda_{k}\left(\cdot \right)$, for example, $\Lambda_{k}\left(s\right)=s$. Let $\delta_{k}>0$ be the triggering threshold. The binary growth indicator $\sigma_{k}\left(t\right)\in \left\{0,1\right\}$ is defined as

(63) $$\sigma_{k}\left(t\right)=\left\{\begin{array}{cc} 1, & t\in \left[t_{0}^{k},t_{1}^{k}\right),\;\Lambda_{k}\left(∥\eta_{k}\left(t_{0}^{k}\right)∥\right)\geq \delta_{k},\\ 1, & t\in \left[t_{j}^{k},t_{j+1}^{k}\right),\;j\geq 1,\;\Lambda_{k}\;\left(\underset{\tau \in [t_{j-1}^{k},t_{j}^{k})}{\sup}∥\eta_{k}\left(\tau \right)∥\right)\geq \delta_{k},\\ 0, & \mathrm{otherwise}. \end{array}\right.$$

Furthermore, select a bounded and integrable nominal growth rate function $r_{k}\left(t,\iota_{k}\right)$ with period $\iota_{k}$ and define the actual growth rate using a saturation operator as

(64) $${\dot{k}}_{2}\left(t\right)=\sigma_{k}\left(t\right){\mathrm{sat}}_{{\bar{r}}_{k}}\left(r_{k}\left(t,\iota_{k}\right)\right),$$

where

(65) $${\mathrm{sat}}_{{\bar{r}}_{k}}\left(s\right)=\left\{\begin{array}{cc} 0, & s<0,\\ s, & 0\leq s\leq {\bar{r}}_{k},\\ {\bar{r}}_{k}, & s>{\bar{r}}_{k}. \end{array}\right.$$

Thus,

(66) $$0\leq {\dot{k}}_{2}\left(t\right)\leq {\bar{r}}_{k},\;k_{2}\left(t\right)\geq k_{20},\;\forall t\geq 0.$$

One can choose $r_{k}\left(t,\iota_{k}\right)=r_{0}$ with $0<r_{0}\leq {\bar{r}}_{k}$. In each activated growth interval, the gain increases along a continuous ramp with a slope not exceeding ${\bar{r}}_{k}$. If growth is activated in the *j*th monitoring interval, the maximum gain increment in that interval satisfies

(67) $$\Delta k_{2,j}=\int_{t_{j}^{k}}^{t_{j+1}^{k}}{\dot{k}}_{2}\left(\tau \right)d\tau \leq {\bar{r}}_{k}\iota_{k}.$$

Compared with direct jump updates, (64) guarantees continuous variation of the dynamic gain and limits its variation per unit time.

**Remark** **5.** *The monitoring vector $\eta_{k}\left(t\right)={\left[\xi_{1}\left(t\right),\;\eta_{2}\left(t\right)\right]}^{T}$ consists of the normalized suspension gap error and vertical velocity, where $|\xi_{1}\left(t\right)|<1$ and $|\eta_{2}\left(t\right)|<1$ represent the corresponding prescribed performance constraints. These dimensionless variables directly characterize the remaining safety margins of the two performance layers, allowing the dynamic gain to increase when either constraint approaches its boundary. Meanwhile, the saturation function limits the growth rate of $k_{2}\left(t\right)$, preventing abrupt control variations and suppressing excessive coil current surges that may adversely affect the suspension electromagnet and its drive circuit.*

### 3.4. Closed-Loop Stability Analysis

**Theorem** **2.** *Under Assumptions 1 and 2, by adopting the extended state observer (14), the adaptive law (25), the fault-tolerant control law (54), the dynamic event-triggered mechanism (57) and the rate-constrained dynamic gain update law (64), it is guaranteed that all closed-loop signals are uniformly ultimately bounded, Zeno behavior is excluded from the dynamic event-triggered mechanism, and constraints (1)–(3) in the control objectives are fulfilled, provided that the design parameters satisfy*

(68) $$k_{1}>\frac{2\alpha_{M}}{\left({\bar{\delta }}_{1}+{\underline{\delta }}_{1}\right)\rho_{1\infty }},\;k_{20}>\frac{{\bar{W}}_{M}}{\rho_{2\infty }},$$

*with ${\bar{W}}_{M}=\rho_{r}{\hat{\theta }}_{\mu \max}\phi_{M}+{\hat{\theta }}_{\mu \max}f_{M}\left(\Phi_{M}u_{M}+2\bar{e}\right)+R_{M}$.*

**Proof.**  Step 1: Proof of constraints (1) and (2).

From the barrier function (51), the virtual controller (53) and (52), one has

(69) $${\dot{V}}_{1}=\frac{2\xi_{1}\left(x_{2}-\alpha_{1}\right)}{\left({\bar{\delta }}_{1}+{\underline{\delta }}_{1}\right)\rho_{1}\left(1-\xi_{1}^{2}\right)}-k_{1}\frac{\xi_{1}^{2}}{1-\xi_{1}^{2}}.$$

Construct the logarithmic barrier function

(70) $$V_{2}=\frac{1}{2}\ln\frac{1}{1-\eta_{2}^{2}}.$$

From the system dynamics and the observation error definitions, one obtains

(71) $${\dot{x}}_{2}={\hat{\theta }}_{\mu }\phi \left(x_{1},u,r_{d}\right)+g+{\hat{x}}_{3}+R\left(t\right),$$

where

(72) $$R\left(t\right)={\tilde{x}}_{3}-{\tilde{\theta }}_{\mu }\phi \left(x_{1},u,r_{d}\right)$$

represents the residual term caused by the extended state estimation error and the equivalent gain estimation error. Thus,

(73) $${\dot{V}}_{2}=\frac{\eta_{2}}{\rho_{2}\left(1-\eta_{2}^{2}\right)}\left({\dot{x}}_{2}-\eta_{2}{\dot{\rho }}_{2}\right).$$

Substituting $u\left(t\right)=v\left(t\right)-e_{u}\left(t\right)$ into the regression function $\phi \left(x_{1},u,r_{d}\right)=bf\left(x_{1},r_{d}\right)u$ gives

(74) $$\phi \left(x_{1},u,r_{d}\right)=\phi_{v}\left(t\right)-bf\left(x_{1},r_{d}\right)e_{u}\left(t\right),$$

where $\phi_{v}\left(t\right)=bf\left(x_{1},r_{d}\right)v\left(t\right)$. Combining this with (59) yields

(75) $${\hat{\theta }}_{\mu }\phi \left(x_{1},u,r_{d}\right)=\left(1+\rho_{r}\right){\hat{\theta }}_{\mu }\phi^{*}\left(t\right)+{\hat{\theta }}_{\mu }\bar{e}bf\left(x_{1},r_{d}\right)\mathrm{sgn}\left(x_{2}\right)-{\hat{\theta }}_{\mu }bf\left(x_{1},r_{d}\right)e_{u}\left(t\right),$$

where $\phi^{*}\left(t\right)=bf\left(x_{1},r_{d}\right)u^{*}\left(t\right)$. Substituting the ideal control input (54) gives the vertical velocity dynamics with event triggering error and observation residual as

(76) $${\dot{x}}_{2}=\eta_{2}{\dot{\rho }}_{2}-k_{2}\left(t\right)\rho_{2}\eta_{2}-\frac{2\rho_{2}^{2}\left(1-\eta_{2}^{2}\right)}{\left({\bar{\delta }}_{1}+{\underline{\delta }}_{1}\right)\rho_{1}\left(1-\xi_{1}^{2}\right)}\xi_{1}-\kappa_{f}\mathrm{sgn}\left(\eta_{2}\right)+W\left(t\right)+R\left(t\right),$$

where

(77) $$W\left(t\right)=\rho_{r}{\hat{\theta }}_{\mu }\phi^{*}\left(t\right)+{\hat{\theta }}_{\mu }\bar{e}bf\left(x_{1},r_{d}\right)\mathrm{sgn}\left(x_{2}\right)-{\hat{\theta }}_{\mu }bf\left(x_{1},r_{d}\right)e_{u}\left(t\right).$$

Thus,

(78) $${\dot{V}}_{2}=-k_{2}\left(t\right)\frac{\eta_{2}^{2}}{1-\eta_{2}^{2}}-\frac{2\xi_{1}x_{2}}{\left({\bar{\delta }}_{1}+{\underline{\delta }}_{1}\right)\rho_{1}\left(1-\xi_{1}^{2}\right)}-\frac{\kappa_{f}\left|\eta_{2}\right|}{\rho_{2}\left(1-\eta_{2}^{2}\right)}+\frac{\eta_{2}}{\rho_{2}\left(1-\eta_{2}^{2}\right)}\left(W(t)+R(t)\right).$$

It can be seen that the coupling term caused by $x_{2}$ in (69) cancels the corresponding term in (78). This cancellation is the key to the unified analysis of the two-layer prescribed performance design and the event-triggered implementation. Because the actual gap has a positive lower bound and (27) holds, there exist constants $\phi_{M}>0$, $f_{M}>0$, ${\hat{\theta }}_{\mu \max}>0$ and $\Phi_{M}\in \left(0,1\right)$ such that

(79) $$|\phi^{*}\left(t\right)|\;\leq \phi_{M},\;|bf(x_{1},r_{d})|\;\leq f_{M},\;|{\hat{\theta }}_{\mu }\left(t\right)|\;\leq {\hat{\theta }}_{\mu \max},\;\Phi \left(|v(t)|\right)\leq \Phi_{M}.$$

From the event triggering condition, in any triggering interval, one has

(80) $$|e_{u}\left(t\right)\left|<\Phi (|v(t)|)|u(t)\right|+\bar{e}.$$

Combining $u\left(t\right)=v\left(t\right)-e_{u}\left(t\right)$ gives

(81) $$|e_{u}\left(t\right)|\;\leq \Phi_{M}u_{M}+\bar{e}.$$

Therefore, from (77), choose

(82) $$W_{M}=\rho_{r}{\hat{\theta }}_{\mu \max}\phi_{M}+{\hat{\theta }}_{\mu \max}f_{M}\left(\Phi_{M}u_{M}+2\bar{e}\right)$$

which ensures $\left|W(t)\right|\leq W_{M}$. Meanwhile, by Theorem 1 and the projection-based adaptive law, ${\tilde{x}}_{3}$ and ${\tilde{\theta }}_{\mu }$ are bounded. Therefore, there exists a constant $R_{M}>0$ such that

(83) $$\left|R(t)\right|\leq R_{M}.$$

Define

(84) $${\bar{W}}_{M}=W_{M}+R_{M},$$

Then,

(85) $$\left|W(t)+R(t)\right|\leq {\bar{W}}_{M}.$$

Choose the Lyapunov function

(86) $$V=V_{o}+V_{1}+V_{2}.$$

By Theorem 1, there exist $\lambda_{o}>0$ and $\sigma_{o}>0$ such that

(87) $${\dot{V}}_{o}\leq -\lambda_{o}V_{o}+\sigma_{o}.$$

Combining (69) and (78) and using the cancellation of the coupling terms in the two-layer barrier functions, one obtains

(88) $$\begin{array}{cc} \dot{V}\leq & -\lambda_{o}V_{o}-k_{1}\frac{\xi_{1}^{2}}{1-\xi_{1}^{2}}-k_{2}\left(t\right)\frac{\eta_{2}^{2}}{1-\eta_{2}^{2}}+\sigma_{o}-\frac{2\xi_{1}\alpha_{1}}{\left({\bar{\delta }}_{1}+{\underline{\delta }}_{1}\right)\rho_{1}\left(1-\xi_{1}^{2}\right)}\\ \; & +\frac{\eta_{2}}{\rho_{2}\left(1-\eta_{2}^{2}\right)}\left(W(t)+R(t)\right). \end{array}$$

In the performance domain, $x_{1d}$, ${\dot{x}}_{1d}$, $\rho_{1}$, ${\dot{\rho }}_{1}$, $\eta_{1}$ and $\xi_{1}$ are bounded. Hence, there exists $\alpha_{M}>0$ such that $|\alpha_{1}|\;\leq \alpha_{M}$. It follows that

(89) $$|\frac{2\xi_{1}\alpha_{1}}{\left({\bar{\delta }}_{1}+{\underline{\delta }}_{1}\right)\rho_{1}\left(1-\xi_{1}^{2}\right)}|\;\leq \frac{2\alpha_{M}}{\left({\bar{\delta }}_{1}+{\underline{\delta }}_{1}\right)\rho_{1\infty }}\frac{\xi_{1}^{2}}{1-\xi_{1}^{2}}+\frac{\alpha_{M}}{\left({\bar{\delta }}_{1}+{\underline{\delta }}_{1}\right)\rho_{1\infty }},$$

Since $\rho_{2}\left(t\right)\geq \rho_{2\infty }$ and $\left|W(t)+R(t)\right|\leq {\bar{W}}_{M}$, one has

(90) $$\frac{\eta_{2}}{\rho_{2}\left(1-\eta_{2}^{2}\right)}\left(W(t)+R(t)\right)\leq \frac{{\bar{W}}_{M}}{\rho_{2\infty }}\frac{\eta_{2}^{2}}{1-\eta_{2}^{2}}+\frac{{\bar{W}}_{M}}{2\rho_{2\infty }}.$$

Substituting (89) and (90) into (88) yields

(91) $$\dot{V}\leq -\lambda_{o}V_{o}-\left(k_{1}-\frac{2\alpha_{M}}{\left({\bar{\delta }}_{1}+{\underline{\delta }}_{1}\right)\rho_{1\infty }}\right)\frac{\xi_{1}^{2}}{1-\xi_{1}^{2}}-{\bar{k}}_{2}\left(t\right)\frac{\eta_{2}^{2}}{1-\eta_{2}^{2}}+C_{e},$$

where

(92) $${\bar{k}}_{2}\left(t\right)=k_{2}\left(t\right)-\frac{{\bar{W}}_{M}}{\rho_{2\infty }},\;C_{e}=\sigma_{o}+\frac{\alpha_{M}}{\left({\bar{\delta }}_{1}+{\underline{\delta }}_{1}\right)\rho_{1\infty }}+\frac{{\bar{W}}_{M}}{2\rho_{2\infty }}.$$

From (66) and the gain condition (68), one has

(93) $${\bar{k}}_{2}\left(t\right)\geq {\bar{k}}_{20}=k_{20}-\frac{{\bar{W}}_{M}}{\rho_{2\infty }}>0.$$

From the definitions of the logarithmic barrier functions (51) and (70), For any s∈[0,1), consider

(94) $$q\left(s\right)=\frac{s}{1-s}+\ln\left(1-s\right).$$

Its derivative is

(95) $$q^{\prime }\left(s\right)=\frac{s}{{\left(1-s\right)}^{2}}\geq 0,$$

and q(0)=0. Hence, q(s)≥0, which gives

(96) $$\frac{s}{1-s}\geq -\ln\left(1-s\right),\;0\leq s<1.$$

Substituting $s=\xi_{1}^{2}$ and $s=\eta_{2}^{2}$, respectively, yields

(97) $$\frac{\xi_{1}^{2}}{1-\xi_{1}^{2}}\geq -\ln\left(1-\xi_{1}^{2}\right)=2V_{1},$$

(98) $$\frac{\eta_{2}^{2}}{1-\eta_{2}^{2}}\geq -\ln\left(1-\eta_{2}^{2}\right)=2V_{2}.$$

Therefore, there exists

(99) $$\lambda_{d}=\min\left\{\lambda_{o},2\left(k_{1}-\frac{2\alpha_{M}}{\left({\bar{\delta }}_{1}+{\underline{\delta }}_{1}\right)\rho_{1\infty }}\right),2{\bar{k}}_{20}\right\}>0$$

such that

(100) $$\dot{V}\leq -\lambda_{d}V+C_{e}.$$

Solving this inequality gives

(101) $$V\left(t\right)\leq V\left(0\right)e^{-\lambda_{d}t}+\frac{C_{e}}{\lambda_{d}}\left(1-e^{-\lambda_{d}t}\right),\;\forall t\geq 0.$$

Therefore, $V_{o}$, $V_{1}$ and $V_{2}$ are uniformly ultimately bounded, and hence, ε, ${\tilde{\theta }}_{\mu }$, $\xi_{1}$ and $\eta_{2}$ are uniformly ultimately bounded. Since boundedness of $V_{1}$ implies $|\xi_{1}\left(t\right)|<1$, it follows from (48) that

(102) $$-{\underline{\delta }}_{1}<\eta_{1}\left(t\right)<{\bar{\delta }}_{1},$$

Thus, $-{\underline{\delta }}_{1}\rho_{1}\left(t\right)<e_{1}\left(t\right)<{\bar{\delta }}_{1}\rho_{1}\left(t\right)$. Similarly, boundedness of $V_{2}$ implies that $|\eta_{2}\left(t\right)|<1$ and consequently $|x_{2}\left(t\right)|<\rho_{2}\left(t\right)$.

Step 2: Proof of uniform ultimate boundedness of all closed-loop signals and verification of constraint (3).

Next, the boundedness of the dynamic gain is proved. From (64), $k_{2}\left(t\right)$ is nondecreasing, and its growth rate is limited. When the monitoring vector exceeds the threshold in a sampling interval, $\sigma_{k}\left(t\right)=1$ and the gain grows in the next sampling interval with a limited slope. From (91), increasing $k_{2}\left(t\right)$ only strengthens the dissipative term $-{\bar{k}}_{2}\left(t\right)\frac{\eta_{2}^{2}}{1-\eta_{2}^{2}}$ in the barrier function $V_{2}$. If growth intervals occurred infinitely often, then $k_{2}\left(t\right)$ would keep increasing, which would drive $\eta_{2}$ and the monitoring vector $\eta_{k}\left(t\right)$ into the threshold set, contradicting infinite growth activation. Hence, the number of activated growth intervals is finite, denoted by $N_{k}$. Since each growth interval has length $\iota_{k}$ and $0\leq {\dot{k}}_{2}\left(t\right)\leq {\bar{r}}_{k}$, one obtains

(103) $$k_{2}\left(t\right)\leq k_{20}+N_{k}{\bar{r}}_{k}\iota_{k}≜k_{2\max}<\infty ,\;\forall t\geq 0.$$

Take $T_{k}=\left(N_{k}+1\right)\iota_{k}$. Then, no new growth interval appears for $t\geq T_{k}$, and thus, $\sigma_{k}\left(t\right)=0$ and ${\dot{k}}_{2}\left(t\right)=0$. Since ${\hat{\theta }}_{\mu }$ is bounded by projection, $f(x_{1},r_{d})$ is bounded and nonsingular under the positive gap lower bound, the ESO error is bounded, the performance variables are bounded and $k_{2}\left(t\right)$ is bounded, so it follows that $u^{*}\left(t\right)$, v(t) and u(t) are uniformly ultimately bounded. Therefore, all closed-loop signals are uniformly ultimately bounded. Furthermore, from (66), one has

(104) $$0\leq {\dot{k}}_{2}\left(t\right)\leq {\bar{r}}_{k},\;k_{2}\left(t\right)\geq k_{20},\;\forall t\geq 0,$$

Therefore, constraint (3) is satisfied.

Step 3: Proof that the dynamic event triggering mechanism is free of Zeno behavior.

First, consider the dynamic gain update sequence. Gain monitoring occurs only at the fixed sampling instants $t_{j}^{k}=j\iota_{k}$, and adjacent monitoring instants satisfy $t_{j+1}^{k}-t_{j}^{k}=\iota_{k}>0$. From the boundedness proof of the dynamic gain above, the number of growth intervals is finite. Therefore, infinitely many dynamic gain updates cannot occur within a finite time.

Next, consider the control input event triggering sequence. The sign switching trigger is caused by the zero crossing of $x_{2}\left(t\right)$. Since the closed-loop state $x_{2}\left(t\right)$ is continuously differentiable and the closed-loop signals, $k_{2}\left(t\right)$ and ${\dot{k}}_{2}\left(t\right)$, are all bounded, ${\dot{x}}_{2}\left(t\right)$ is bounded. Therefore, $x_{2}\left(t\right)$ cannot generate infinitely many effective sign switches within a finite time. Let $T_{1}>0$ denote a lower bound on the minimum interval between any two adjacent sign switching triggers.

In an adjacent dynamic threshold triggering interval $\left[t_{i},t_{i+1}\right)$ without sign switching, the control input is held constant by a zero-order hold. Therefore,

(105) $${\dot{e}}_{u}\left(t\right)=\dot{v}\left(t\right).$$

From the boundedness results above, the closed-loop signals are bounded, $k_{2}\left(t\right)$ is bounded and $0\leq {\dot{k}}_{2}\left(t\right)\leq {\bar{r}}_{k}$. Hence, there exists a constant $L_{v}^{*}>0$ such that

(106) $$|\dot{v}\left(t\right)|\;\leq L_{v}^{*},\;t\in \left[t_{i},t_{i+1}\right).$$

where $L_{v}^{*}$ can be chosen as an upper bound that includes the influence of the dynamic gain variation rate, for example,

(107) $$L_{v}^{*}=L_{v0}+\left(1+\rho_{r}\right)\frac{{\bar{r}}_{k}\rho_{2M}}{\theta_{\mu \min}f_{\min}},$$

Here, $L_{v0}$ denotes the upper bound of the continuous part of the derivative excluding the ${\dot{k}}_{2}\left(t\right)$ term, $\rho_{2M}=\sup_{t\geq 0}\rho_{2}\left(t\right)$ and $f_{\min}$ denotes the positive lower bound of $|bf(x_{1},r_{d})|$ on the reachable compact set. Since $e_{u}\left(t_{i}\right)=0$, one has

(108) $$|e_{u}\left(t\right)|\;\leq L_{v}^{*}\left(t-t_{i}\right).$$

When a dynamic threshold trigger occurs, it must hold that

(109) $$|e_{u}\left(t_{i+1}\right)\left|=\Phi (\right|v(t_{i+1})\left|)\right|u(t_{i+1})|+\bar{e}\geq \bar{e},$$

Therefore,

(110) $$t_{i+1}-t_{i}\geq \frac{\bar{e}}{L_{v}^{*}}>0.$$

Consequently, choose

(111) $$T_{\min}^{*}=\min\left\{T_{1},\frac{\bar{e}}{L_{v}^{*}},\iota_{k}\right\}>0,$$

which implies that all inter-event intervals have a strictly positive lower bound. Hence, the closed-loop system is free of Zeno behavior. In summary, constraints (1) and (2), uniform ultimate boundedness of all closed-loop signals, constraint (3) and Zeno freeness are all established. This completes the proof. □

## 4. Numerical Simulation

In this section, numerical simulations are presented with the aim of verifying the effectiveness of the proposed two-layer prescribed performance dynamic event-triggered fault-tolerant control method. To compare the proposed scheme with Adaptive control [51], LQR control [52], and fuzzy PID control [53] and verify its adaptability under various loads, the gap error and vertical velocity responses under Δm=200kg, 450kg, and 700kg are displayed in the following comparative simulations. The primary simulation parameters are listed in Table 1.

### 4.1. Simulation of the Proposed Control Strategy

**Figure 2:**
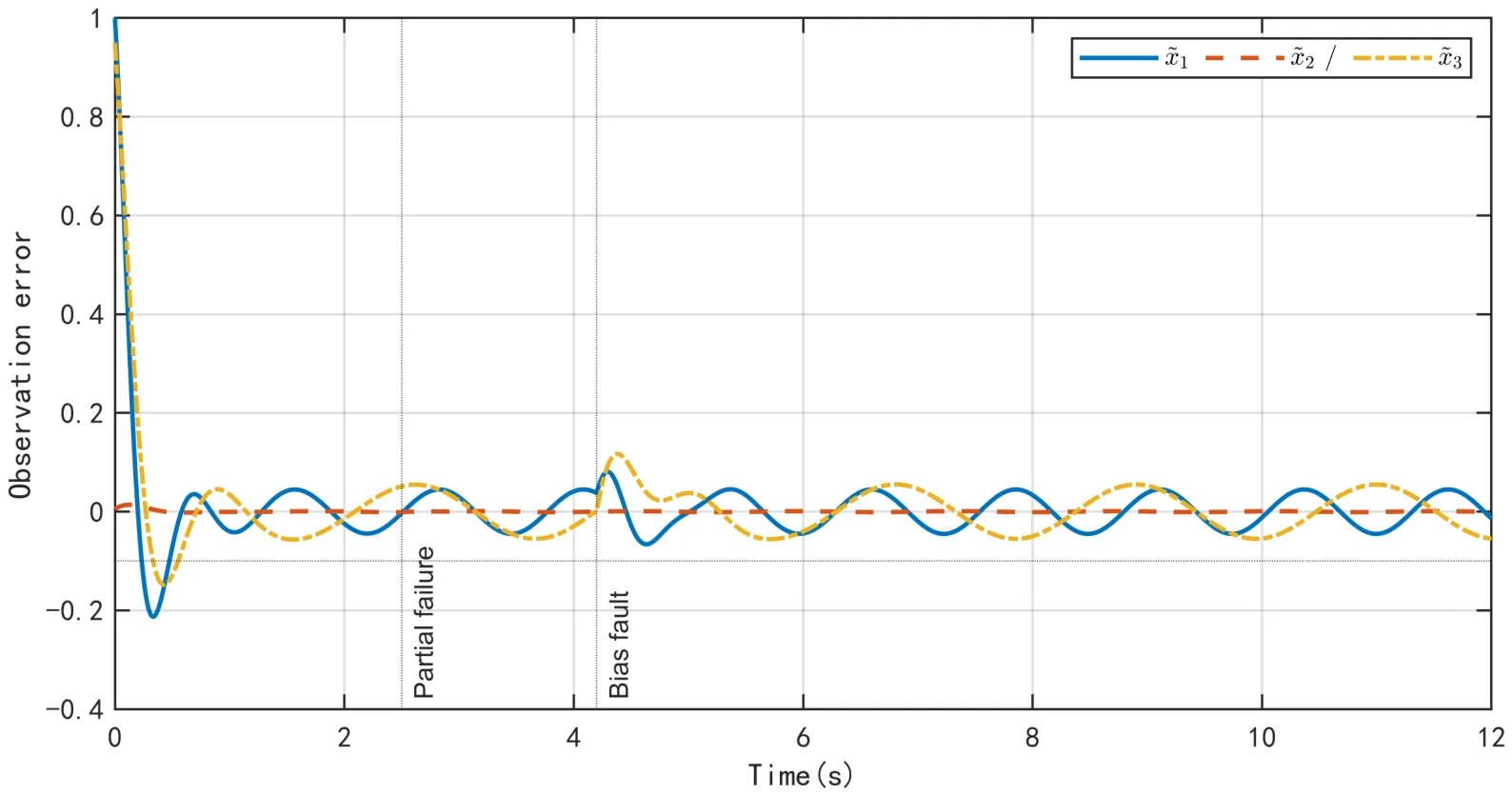
Observation error response curve.

**Figure 3:**
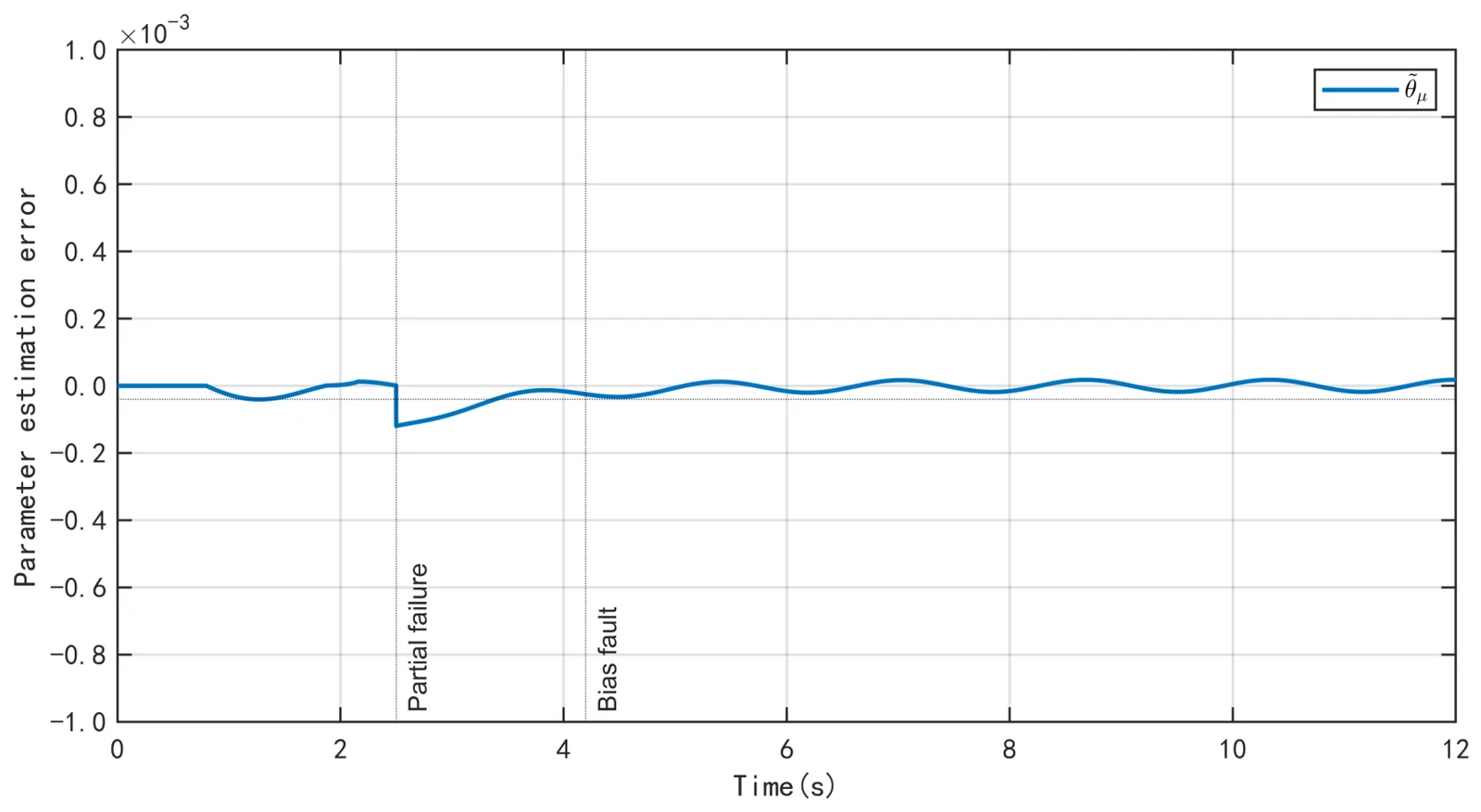
Parameter estimation error response curve.

In the observer-based event-triggering and dynamic gain simulations, a baseline load variation of Δm=200kg is considered. A partial loss of actuator effectiveness and an additive bias fault are introduced at t=2.5 s and t=4.2 s, respectively. Figure 2 and Figure 3 present the corresponding observation and parameter estimation errors. As shown in Figure 2, the observation errors ${\tilde{x}}_{1}$, ${\tilde{x}}_{2}$, and ${\tilde{x}}_{3}$ rapidly converge to a small neighborhood of zero despite the occurrence of actuator faults. Figure 3 shows that the parameter estimation error ${\tilde{\theta }}_{\mu }$ remains uniformly ultimately bounded. These results demonstrate that the proposed projection-based adaptive extended state observer effectively estimates the equivalent unknown control gain caused by load variation and actuator faults while compensating for the lumped disturbance.

**Figure 4:**
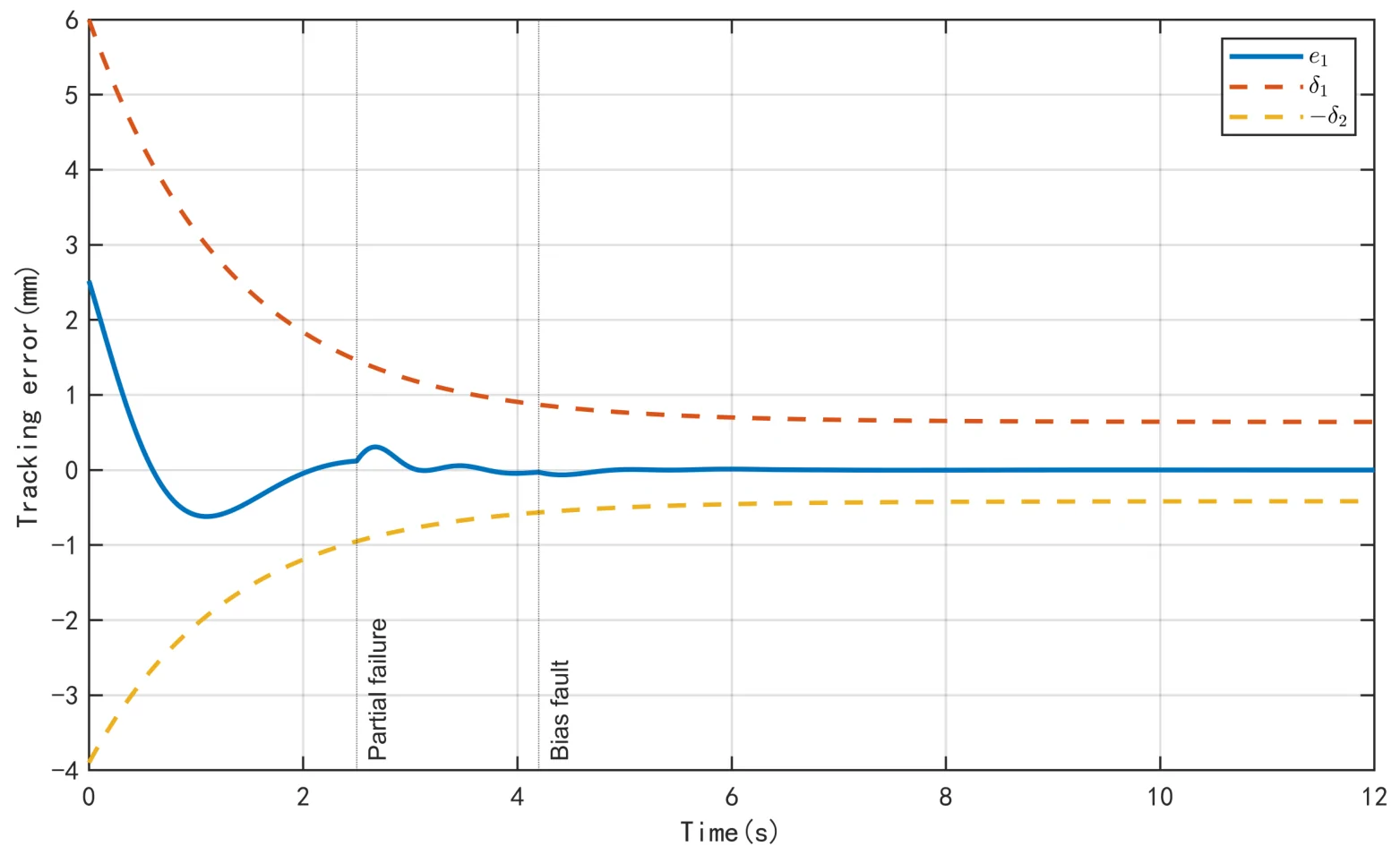
The gap tracking error under the asymmetric prescribed performance constraint.

**Figure 5:**
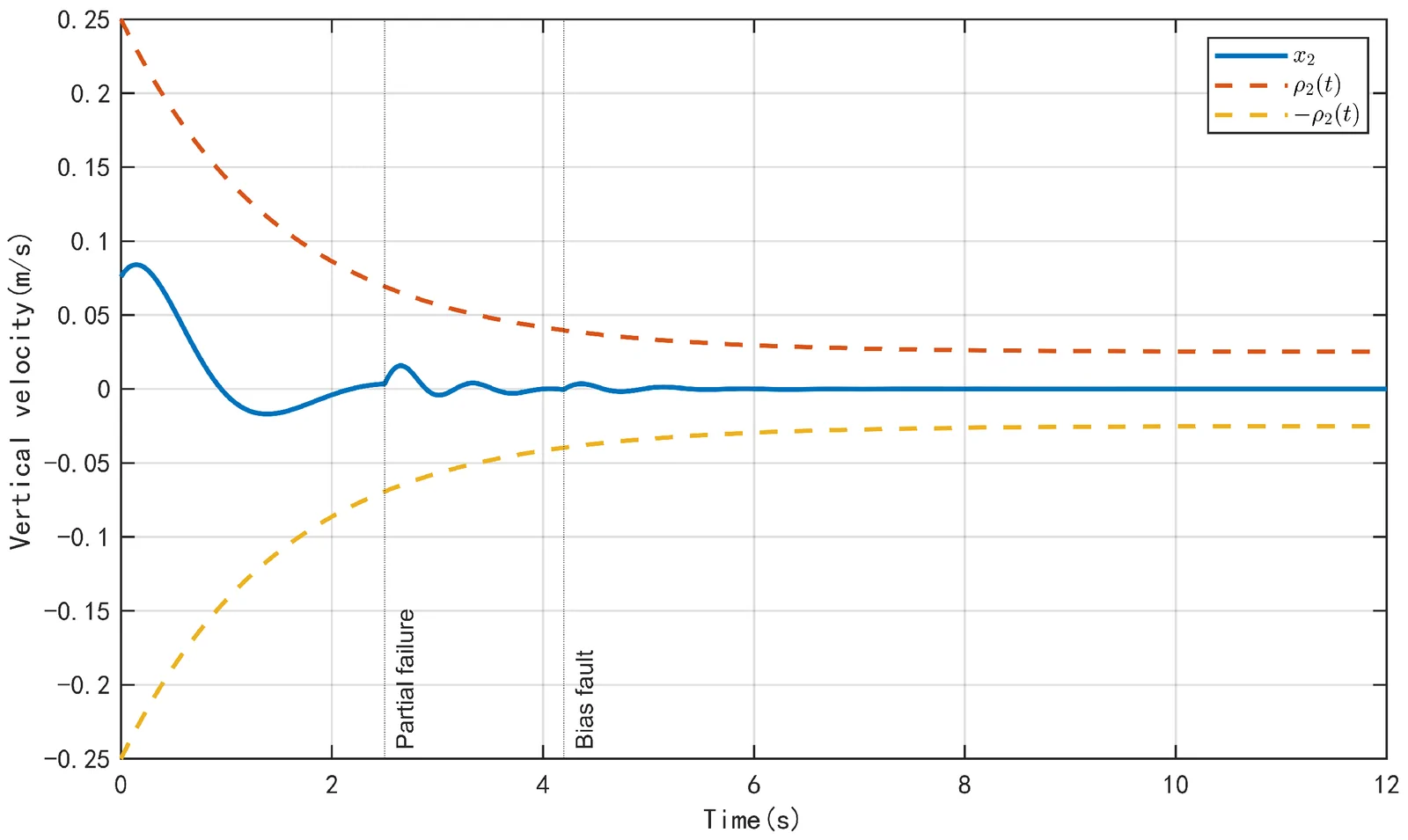
The vertical velocity under the prescribed performance constraint.

As evidenced in Figure 4, the gap tracking error $e_{1}$ remains within the prescribed performance boundaries throughout the simulation, thereby validating the efficacy of the proposed asymmetric error transformation in reflecting the distinct upper and lower safety margins of the suspension gap. As illustrated in Figure 5, the vertical velocity, denoted by $x_{2}$, satisfies the condition $|x_{2}\left(t\right)|<\rho_{2}\left(t\right)$ over the entire time interval under consideration. The proposed method, therefore, enforces the suspension gap constraint while suppressing excessive vertical motion, which is beneficial to both operating safety and ride comfort.

**Figure 6:**
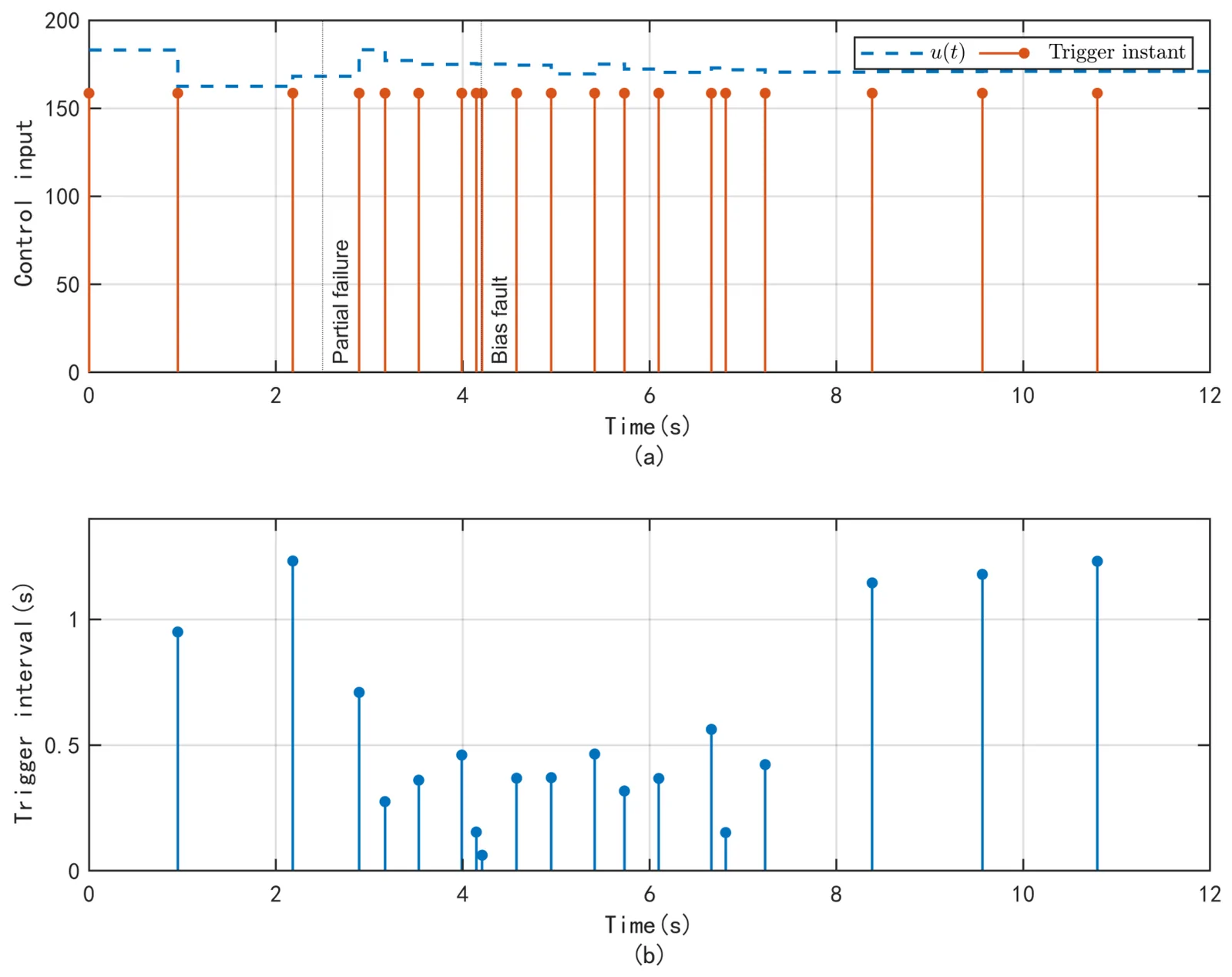
Dynamic event-triggered control results: (**a**) control input and triggering instants; (**b**) inter-event intervals.

As illustrated in Figure 6, the control input and the inter-event intervals under the dynamic event triggering mechanism are demonstrated. The actual control input u(t) is updated through a zero-order hold, and positive intervals are observed between adjacent triggering instants. This indicates the information efficiency of the proposed mechanism. Instead of transmitting control updates at every sampling instant, the controller releases information only when the triggering condition indicates a sufficiently large deviation in control. The positive inter-event intervals shown in Figure 6 therefore reflect the exclusion of Zeno behavior and the reduction in redundant information transfer between the controller and the actuator.

**Figure 7:**
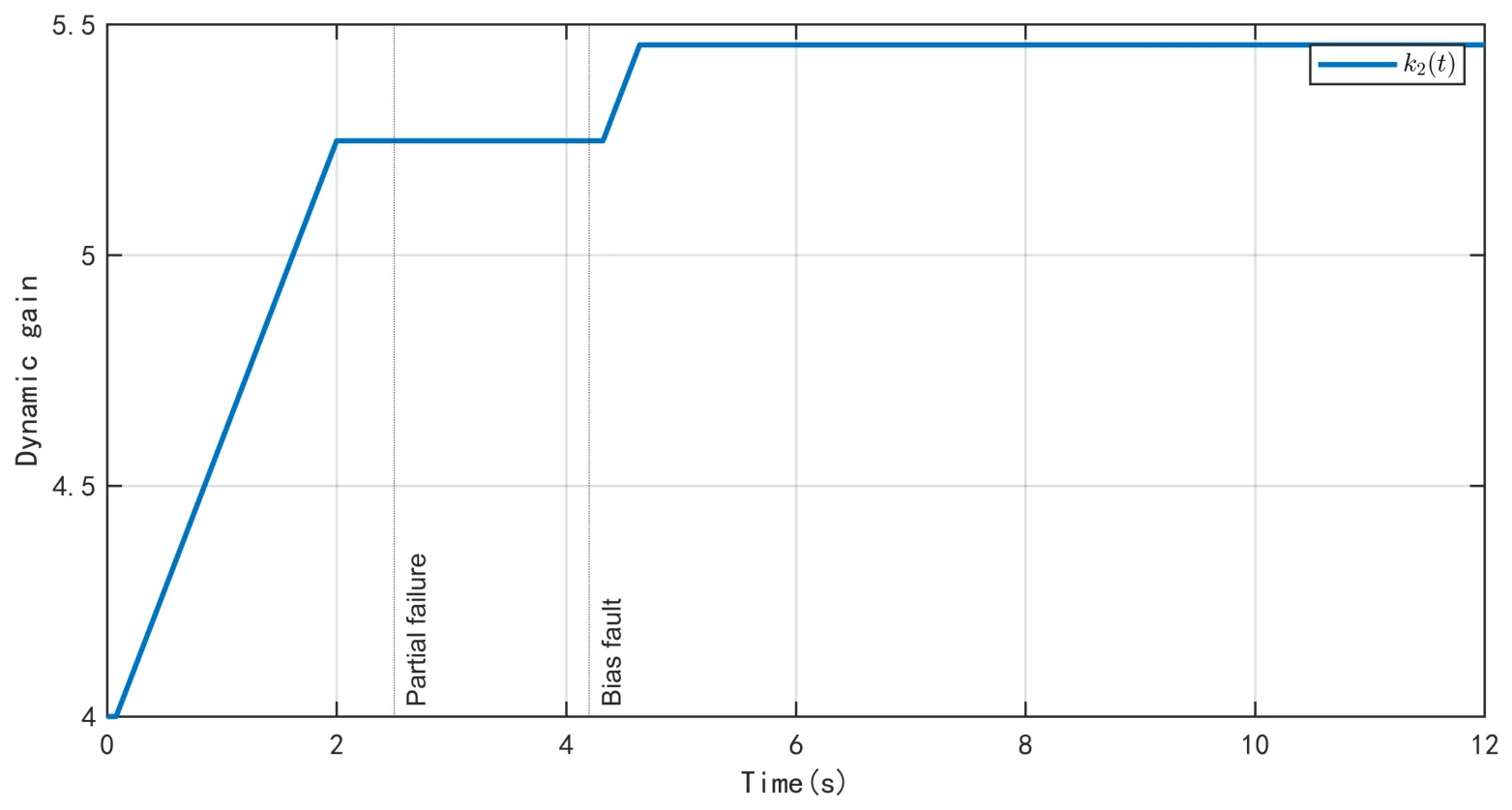
Trajectory of event-triggered dynamic gain.

**Figure 8:**
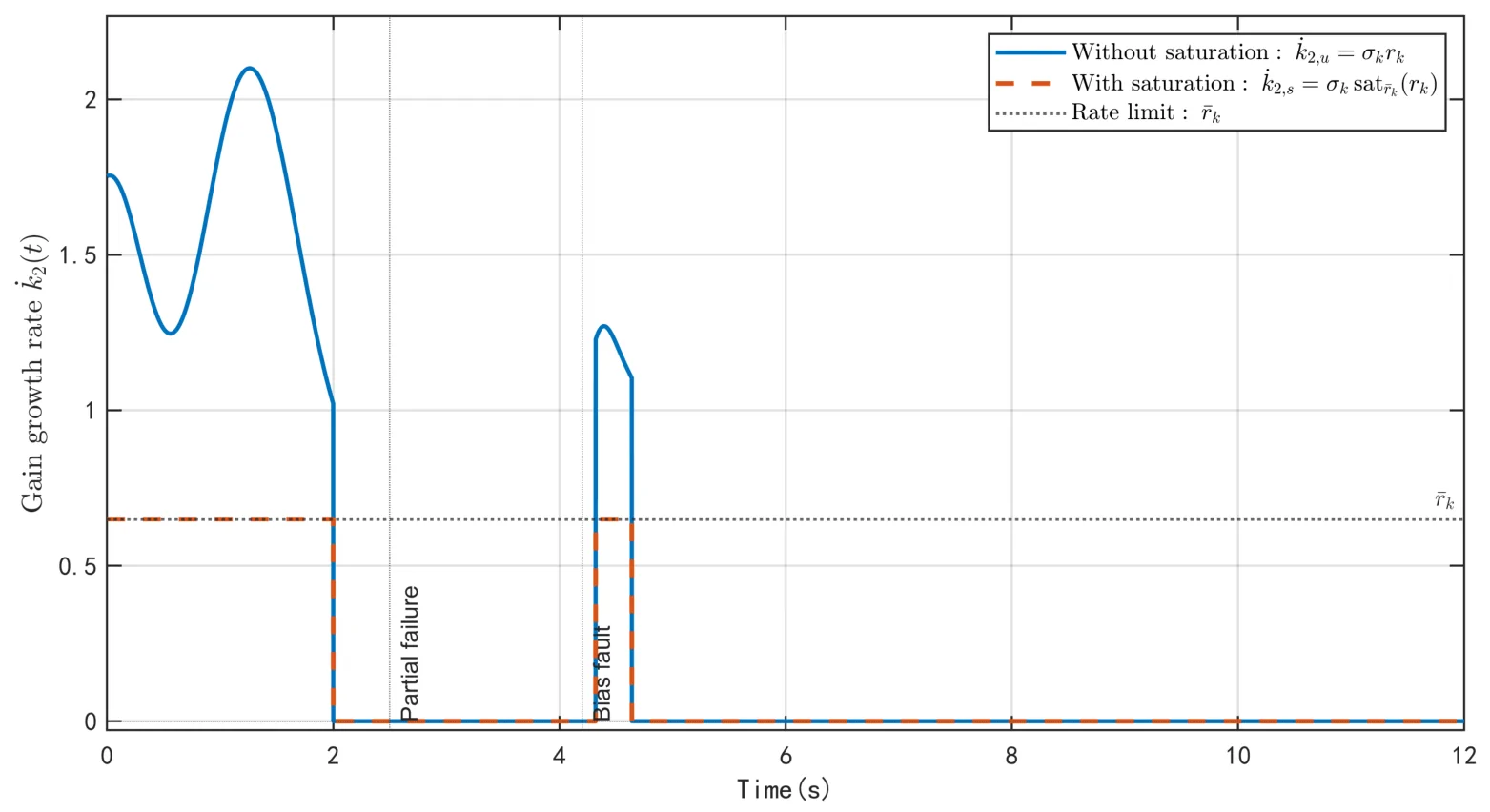
Trajectory of the gain variation rate under the rate constraint.

As shown in Figure 7, the dynamic gain $k_{2}\left(t\right)$ increases when the monitoring condition is activated and remains constant after the system response has stabilized. Figure 8 compares the unconstrained and saturated gain variation rates under the same monitoring signal and nominal growth-rate command. During the active intervals, the unconstrained variation rate exceeds the prescribed limit, whereas the saturated variation rate is restricted to ${\bar{r}}_{k}$; during the inactive intervals, both variation rates are zero. Therefore, the proposed update law guarantees $0\leq {\dot{k}}_{2,s}\left(t\right)\leq {\bar{r}}_{k}$, thereby preventing excessively rapid gain adjustment while retaining the required adaptive capability.

The main design parameters are selected according to the stability conditions and their influence on the closed-loop performance. In particular, the observer bandwidth is chosen to satisfy ω>1/2, and the controller gains $k_{1}$ and $k_{20}$ are selected according to the sufficient conditions in Theorem 2. The remaining parameters are tuned to balance estimation performance, control accuracy, triggering frequency, and smooth gain adjustment. Their main qualitative effects are summarized in Table 2, where +, −, and ⋆ denote positive, negative, and no dominant direct correlation, respectively. These symbols represent general tuning tendencies rather than strict monotonic relationships.

### 4.2. Simulation Comparison with Other Algorithms

In evaluating the proposed strategy, Adaptive control [51], LQR control [52], and Fuzzy PID control [53] are utilized as comparison methods.

**Figure 9:**
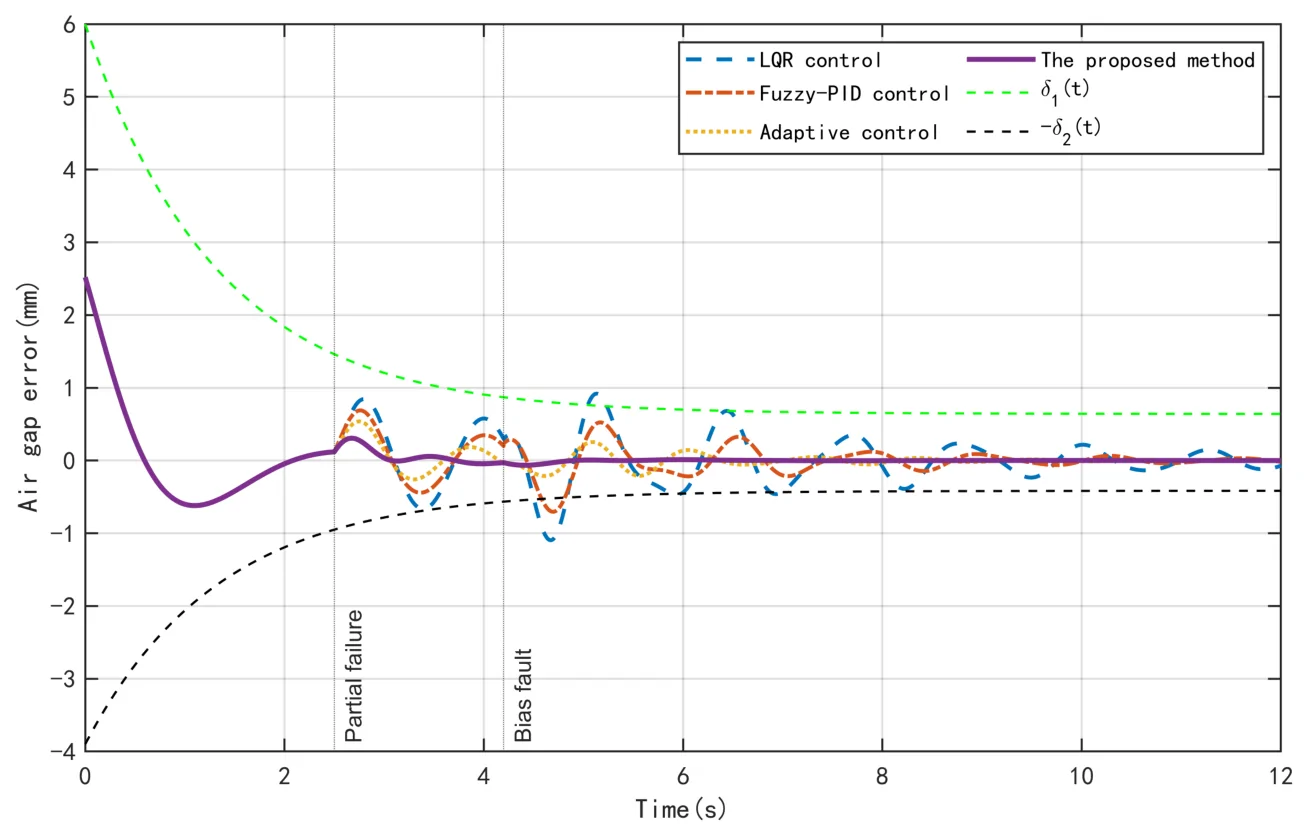
The air gap errors under actuator faults.

As illustrated in Figure 9, following the bias fault at t = 4.2 s, the LQR and Fuzzy-PID errors exceed the lower performance boundary, while the adaptive controller remains within the constraint but exhibits slower convergence and larger oscillations. In contrast, the proposed method keeps the levitation gap error strictly within the asymmetric boundaries throughout the simulation, resulting in smaller transient peaks and faster oscillation attenuation.

**Figure 10:**
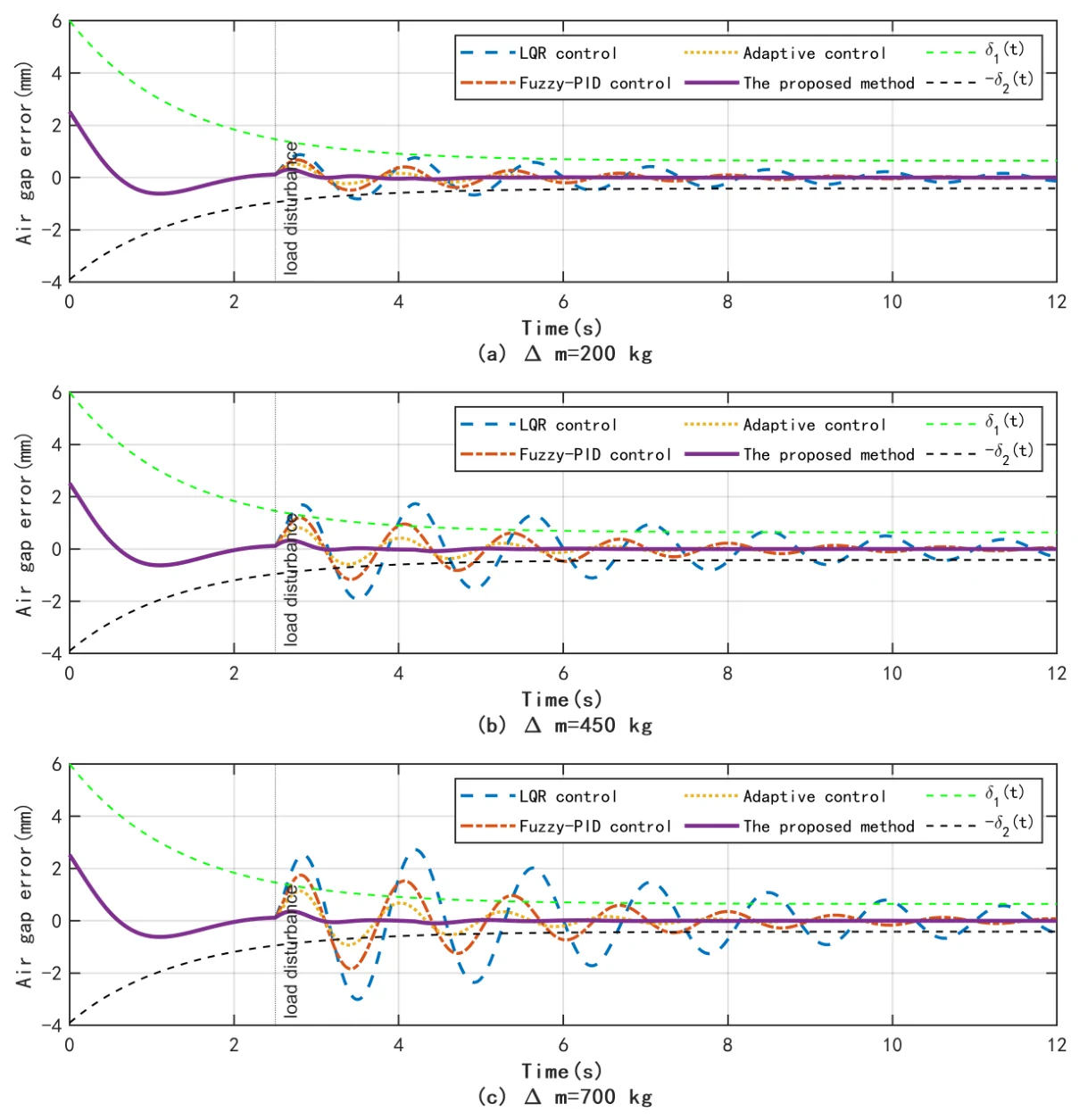
The air gap errors under different loads: (**a**) Δm=200kg; (**b**) Δm=450kg; (**c**) Δm=700kg.

**Figure 11:**
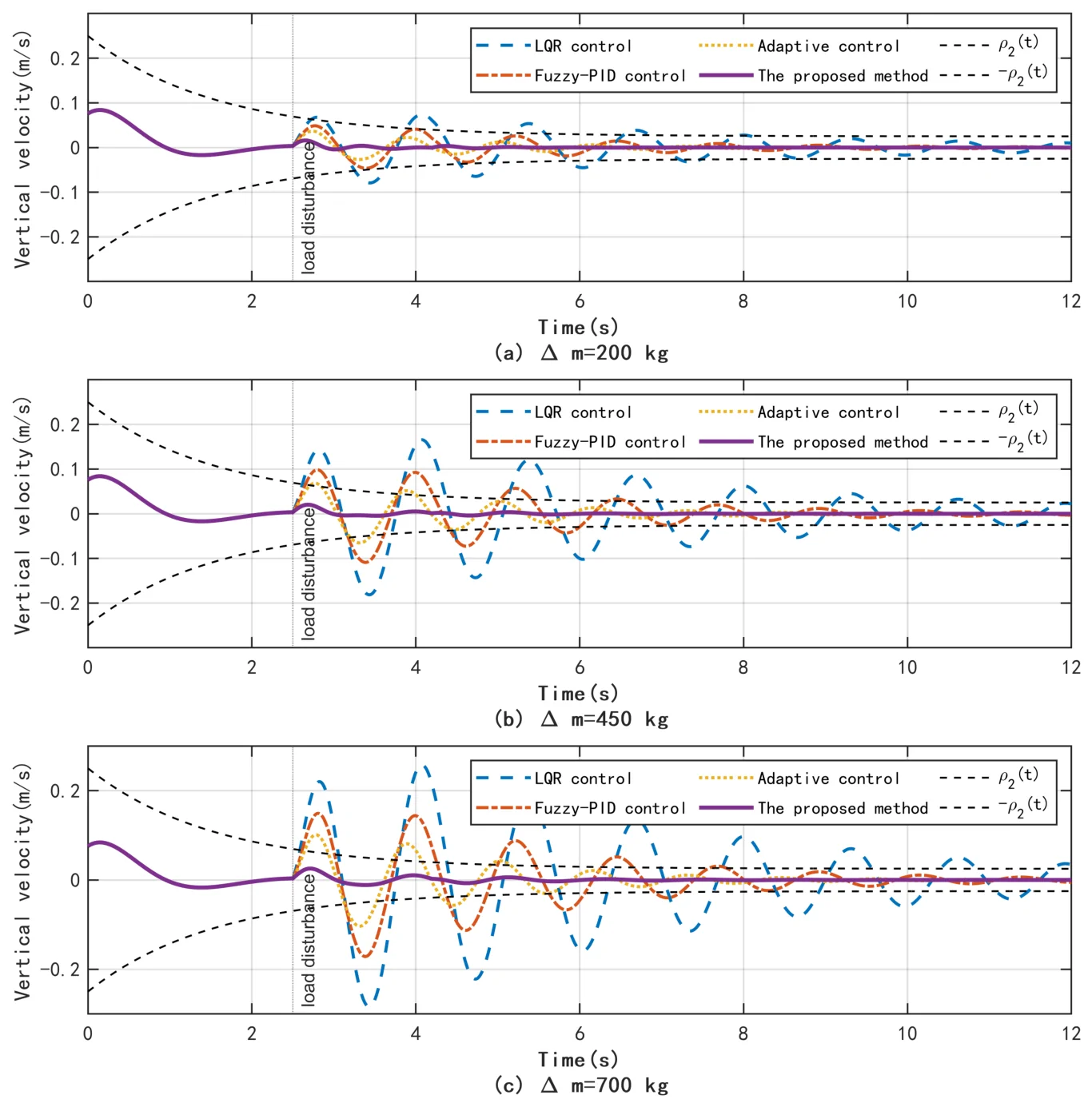
The vertical velocity responses under different loads: (**a**) Δm=200kg; (**b**) Δm=450kg; (**c**) Δm=700kg.

As exhibited in Figure 10 and Figure 11, under the Δm=200kg load, most responses remain within the prescribed boundaries, with only brief violations observed in the LQR method. As the load increases to 450kg and 700kg, the LQR and Fuzzy-PID responses exhibit increasingly pronounced boundary violations and oscillations, while the adaptive controller also fails to maintain the constraints under the largest load. In contrast, the proposed method keeps both the levitation-gap error and vertical velocity strictly within their prescribed bounds under all three load conditions, with smaller peaks and faster convergence.

**Figure 12:**
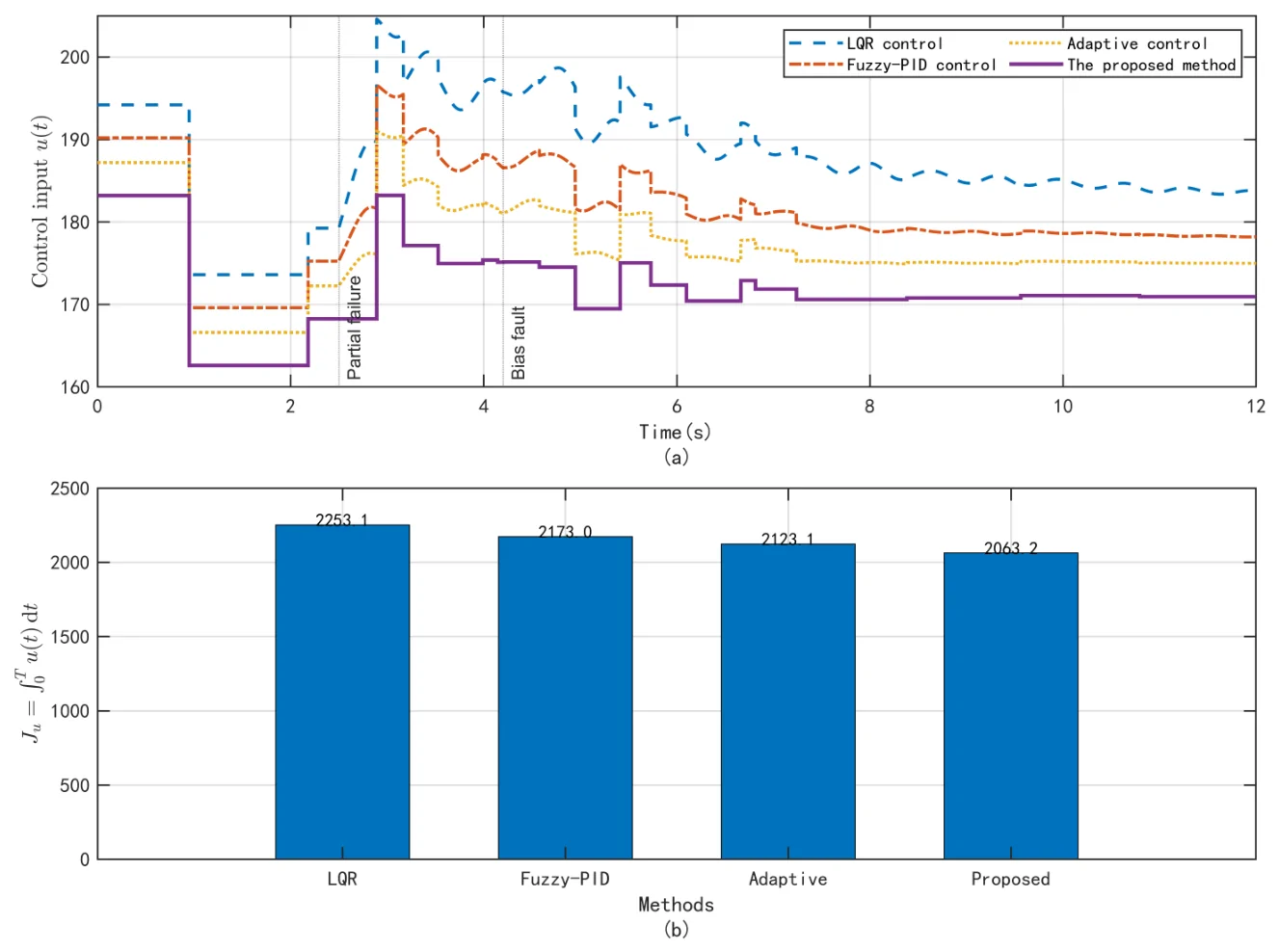
The control inputs and equivalent actuator energy consumption under actuator faults: (**a**) control inputs of different control methods; (**b**) equivalent actuator energy consumption of different control methods.

Figure 12 compares the control inputs of the proposed method, Adaptive control, LQR control, and Fuzzy-PID control under the same operating conditions. Since the control input is defined as $u\left(t\right)=i^{2}\left(t\right)$, the integral

$$J_{u}=\int_{0}^{T}u\left(t\right)\;dt$$

can be used as an equivalent copper-loss index under the same coil resistance. It is observed that the proposed method maintains a lower control input level and yields the smallest equivalent actuator energy index among the compared methods. Specifically, the corresponding index is reduced by 8.4%, 5.1%, and 2.8% compared with LQR control, Fuzzy PID control, and Adaptive control, respectively, while satisfactory suspension performance is still maintained. 

## 5. Conclusions

This paper has addressed the issue of prescribed performance fault-tolerant control for a maglev train suspension system subject to load variations, external disturbances, and actuator faults. The article designs a projection-based adaptive extended state observer. The two-layer prescribed performance framework is further developed to simultaneously enforce asymmetric gap constraints and vertical velocity bounds. Furthermore, a rate-constrained dynamic gain event-triggered mechanism is designed to reduce unnecessary control updates while avoiding abrupt gain variations. Theoretical analysis demonstrates the uniform ultimate boundedness of all closed-loop signals, the preservation of the prescribed constraints, the boundedness of the dynamic gain and its variation rate, and the absence of Zeno behavior. In comparison with LQR, fuzzy PID, and Adaptive control, the proposed method has been shown to provide enhanced transient response, constraint preservation, and disturbance rejection under fault and load disturbance conditions. In future research, the focus will be directed towards actuator saturation and multi-suspension-point coupling. 

## Figures and Tables

**Table 1:** System parameters (physical parameters from [54]).

Parameter	Value	Parameter	Value
Physical parameters and load variation cases from the literature			
*k*	$0.00545\;N\cdot m^{2}/A^{2}$	$m_{0}$	725kg
*R*	4.4Ω	$L_{0}$	0.908H
$\delta_{0}$	0.008m	Δm	200kg
Prescribed performance, event triggering and dynamic gain design parameters of this paper			
$\rho_{10}$	$0.75\delta_{0}=6.0\times 10^{-3}$	$\rho_{1\infty }$	$0.08\delta_{0}=6.4\times 10^{-4}$
$l_{1}$	0.75	${\bar{\delta }}_{1}$	1.00
${\underline{\delta }}_{1}$	0.65	${\bar{r}}_{k}$	0.65
$\rho_{20}$	0.25	$\rho_{2\infty }$	0.025
$l_{2}$	0.65	$\rho_{r}$	1.05
$\bar{e}$	0.05	$\iota_{k}$	0.08
$\delta_{k}$	0.30	$k_{20}$	4.0

**Table 2:** Qualitative influence of the main design parameters.

Category	Parameter	Constraint Accuracy	Response Speed	Trigger Frequency	Control/Gain Variation
Prescribed performance	$\rho_{1\infty },\;\rho_{2\infty }$	−	⋆	⋆	⋆
	$l_{1},\;l_{2}$	⋆	+	⋆	+
Observer	ω	⋆	+	⋆	⋆
	Γ	⋆	+	⋆	⋆
Controller	$k_{1},\;k_{20}$	+	+	⋆	+
Event-triggering	*M*	+	⋆	+	⋆
	$\bar{e}$	−	⋆	−	⋆
Dynamic gain	$\delta_{k}$	⋆	−	⋆	−
	${\bar{r}}_{k}$	⋆	+	⋆	+

## Data Availability

The original contributions presented in this study are included in the article. Further inquiries can be directed to the corresponding author.
